# Novel glycosidase from *Paenibacillus lactis* 154 hydrolyzing the 28-*O*-*β*-d-glucopyranosyl ester bond of oleanane-type saponins

**DOI:** 10.1007/s00253-024-13109-2

**Published:** 2024-04-04

**Authors:** Zongzhan Wu, Wenyu Dou, Xiaolin Yang, Tengfei Niu, Zhuzhen Han, Li Yang, Rufeng Wang, Zhengtao Wang

**Affiliations:** 1 Institute of Chinese Materia Medica, Shanghai University of Traditional Chinese Medicine, Shanghai, 201203 People’s Republic of China; 2 The SATCM Key Laboratory for New Resources and Quality Evaluation of Chinese Medicines, Shanghai University of Traditional Chinese Medicine, Shanghai, 201203 People’s Republic of China; 3 The MOE Key Laboratory for Standardization of Chinese Medicines, Shanghai University of Traditional Chinese Medicine, Shanghai, 201203 People’s Republic of China

**Keywords:** Biotransformation, Glycoside hydrolase family 3, Pseudoginsenoside RP1, Tarasaponin VI, Molecular docking

## Abstract

**Abstract:**

Oleanane-type ginsenosides are a class of compounds with remarkable pharmacological activities. However, the lack of effective preparation methods for specific rare ginsenosides has hindered the exploration of their pharmacological properties. In this study, a novel glycoside hydrolase *Pl*GH3 was cloned from *Paenibacillus lactis* 154 and heterologous expressed in *Escherichia coli*. Sequence analysis revealed that *Pl*GH3 consists of 749 amino acids with a molecular weight of 89.5 kDa, exhibiting the characteristic features of the glycoside hydrolase 3 family. The enzymatic characterization results of *Pl*GH3 showed that the optimal reaction pH and temperature was 8 and 50 °C by using *p*-nitrophenyl-*β*-d-glucopyranoside as a substrate, respectively. The *K*_m_ and *k*_cat_ values towards ginsenoside Ro were 79.59 ± 3.42 µM and 18.52 s^−1^, respectively. *Pl*GH3 exhibits a highly specific activity on hydrolyzing the 28-*O*-*β*-d-glucopyranosyl ester bond of oleanane-type saponins. The mechanism of hydrolysis specificity was then presumably elucidated through molecular docking. Eventually, four kinds of rare oleanane-type ginsenosides (calenduloside E, pseudoginsenoside RP1, zingibroside R1, and tarasaponin VI) were successfully prepared by biotransforming total saponins extracted from *Panax japonicus*. This study contributes to understanding the mechanism of enzymatic hydrolysis of the GH3 family and provides a practical route for the preparation of rare oleanane-type ginsenosides through biotransformation.

**Key points:**

*• The glucose at C-28 in oleanane-type saponins can be directionally hydrolyzed.*

*• Mechanisms to interpret PlGH3 substrate specificity by molecular docking.*

*• Case of preparation of low-sugar alternative saponins by directed hydrolysis.*

**Supplementary information:**

The online version contains supplementary material available at 10.1007/s00253-024-13109-2.

## Introduction

Oleanane-type saponins are highly valued pharmacologically active compounds that found in plants of the *Panax* genus (Yang et al. 2014, 2021). Of particular interest are the rare deglycosylated oleanane-type saponins that exhibit a variety of activities. For example, calenduloside E (CE) has demonstrated significant protection against myocardial ischemia–reperfusion injury (Wang et al. 2020, 2021a), and zingibroside R1 (ZR1) has exhibited notable anti-tumor and anti-angiogenic effects (Zheng et al. 2019).

However, the isolation of rare deglycosylated saponins directly from raw herbal materials of the *Panax* genus presents a substantial challenge due to their limited abundance (Ma et al. 2021). Previous approaches to obtain these valuable saponins have involved in traditional physical extraction, chemical hydrolysis, and biotransformation methods (Kim et al. 2012; Li et al. 2015; Ramirez-Estrada et al. 2016). Remarkably, enzymatic conversion via targeted glycoside hydrolysis has emerged as an ideal and efficient method for selectively removing sugar moieties from saponins. This approach offers several advantages, including eco-friendliness, product specificity, controllable process parameters, and mild reaction conditions (Li et al. 2018; Zhang et al. 2019).

**Fig. 1:**
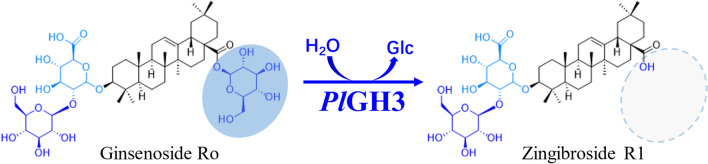
Specific hydrolysis of glucoester groups in ginsenoside Ro by *Pl*GH3

Several enzymes have been identified, recombinantly expressed, and successfully applied to produce target rare dammarane-type saponins (Cui et al. 2011; Quan et al. 2013; Wang et al. 2015). Although various desired rare deglycosylated dammarane-type ginsenosides have been enzymatically prepared on a large scale, the approaches to produce oleanane-type ginsenosides are still rarely reported due to the lack of applicable biocatalysts and suitable substrates. Recent research of chemical chromatographic fingerprint analysis has indicated that the total saponins of *P. japonicus* (TSPJ) were mainly composed of four oleanane-type saponins (Qi et al. 2021), including ginsenoside Ro (Ro), pseudoginsenoside RT1 (RT1), chikusetusaponin IV (C-IV), and chikusetusaponin IVa (C-IVa) (Han et al. 2005; Yoshizaki et al. 2013). These four saponins share a common *β*-d-glucopyranosyl ester bond at the C-28 position in their chemical structures. Consequently, TSPJ presents a suitable substrate to produce four deglycosylated and valuable oleanane-type saponins, namely zingibroside R1 (ZR1), tarasaponin VI (TVI), pseudoginsenoside RP1 (RP1), and calenduloside E (CE), through enzymatic hydrolysis of the glucose moiety. However, there is currently a lack of reported recombinant enzymes that exhibit the essential catalytic function required for this purpose (Fig. 1).

In our previous study, the predicted candidate enzyme cloned from *P. lactis* 154 using G4HIT7-9BACL (WP_007131062.1) as a template gene was selected for investigating its hydrolysis activity on ginsenosides (Wang et al. 2023). However, sequence analysis revealed several amino acid differences in the obtained protein sequence. Although this enzyme did not exhibit the desired activity towards notoginsenoside Fc, it demonstrated efficient and specific hydrolytic conversion characteristics towards TSPJ. Considering its origin from *P*. *lactis* and classification within the GH3 family, we designated this enzyme as *Pl*GH3.

In summary, we investigated, characterized, and employed a novel glycosidase, *Pl*GH3, which specifically hydrolyzes the glucose at the C-28 site in oleanane-type saponins. The hydrolysis specificity was then presumably revealed through molecular docking analysis. *Pl*GH3 was further utilized for the simultaneous enzymatic synthesis of these rare and valuable natural oleanane-type saponins from TSPJ. This study facilitates the production of these four rare natural products, thereby significantly contributing to the future development of promising compounds of high value and rarity.

## Material and methods

### Material

*Paenibacillus lactis* was purchased from DSMZ (www.dsmz.de). *Escherichia coli* DH5α and *E. coli* BL21 (DE3) were used as the host for gene cloning and recombinant protein expression, respectively.

4-Nitrophenyl (*p*NP), 2-nitrophenyl (*o*NP), *p-*nitrophenyl*-β*-d-glucopyranoside (*p*NP-*β*-D-Glc), *o*-nitrophenyl-*β*-d-glucopyranoside (*o*NP-*β*-D-Glc), *p*-nitrophenyl-*β*-d-galactopyranoside (*p*NP-*β*-D-Gal), *o*-nitrophenyl-*β*-d-galactopyranoside (*o*NP-*β*-D-Gal), *p*-nitrophenyl-*β*-d-xylopyranoside (*p*NP-*β*-D-Xyl), and *p*-nitrophenyl-*α*-d-glucopyranoside (*p*NP-*α*-D-Glc) were purchased from Aladdin Biochemical Technology Co., Ltd (Shanghai, China). Ro, RT1, C-IV, C-IVa, ZR1, oleanolic acid, and CE were purchased from the Chengdu Biopurify Phytochemicals Ltd (Chengdu, Sichuan, China). T-VI and RP1 were isolated in this work and determined by MS and NMR. TSPJ were extracted from the rhizomes of *P. japonicus* with 80% ethanol and purified as previous work (He et al. 2017).

Silica gel 60 F254 HPTLC plates were purchased from Yinlong Co., LTD. (Yantai, Shandong, China). HPLC-grade acetonitrile was purchased from Thermo Fisher Technology (China) Co., LTD. (Shanghai, China). Other reagents (analytical grade) were purchased from Sinopharm Chemical Reagent Co., Ltd (Shanghai, China). Plasmid extraction kits, DNA purification kits, and gel extraction kits were purchased from Generay Biotech Co., Ltd. (Shanghai, China). Other biochemical reagents and the SDS-PAGE preparation kit were purchased from Sangon Biotech Co., Ltd. (Shanghai, China).

### Molecular cloning, expression, and purification

The gene encoding glycoside hydrolase was amplified by polymerase chain reaction (PCR) using primers: forward 5′-CGC*GGATCC*ATGAGAAACCATACTTTAGATACG-3′ and reverse 5′-CCC*AAGCTT* TCAGCTTCTACGGTATCTCTTC-3′. The amplified PCR products were digested by the restriction enzyme sites of *Bam*HI and *Hin*dIII and subcloned into the pET-28a vector (Novagen). The plasmid pET-28a-*Pl*GH3 was obtained, and the plasmid sequence information is shown in the supplementary materials (Figure S1).

The plasmid pET-28a-*Pl*GH3 was expressed in *E. coli* BL21 (DE3), grown in LB broth containing 50 µg/mL kanamycin at 37 °C and induced to express recombinant protein by adding isopropyl *β*-d-thiogalactoside (IPTG) to a final concentration of 0.2 mM when the optical density OD_600_ to 0.6. The strains were then incubated at 16 °C for 24 h. The cells were harvested by centrifugation at 5000g for 10 min. The pellets were resuspended in 20 mM sodium phosphate buffer (pH 7.4, 500 mM NaCl) and treated by sonication. After centrifugation at 12,000g for 20 min, the supernatant fraction was used as the crude enzyme. The N-terminal His-tagged fusion protein was executed using a His trap Ni–NTA FF column (GE Healthcare), The specification is 5 mL packing column, and the flow rate is controlled by a peristaltic pump at 5 mL/min. After washing with 25 mL of washing buffer, 15 mL of eluent containing 150 mM imidazole is used to elute the protein of interest. The eluent was concentrated through a 30 kD ultrafiltration tube, and the solvent was replaced with a 20 mM sodium phosphate buffer (pH 7.0) containing 10% glycerol (*v/v*). The expression and purification of *PlGH3* were analyzed by sodium dodecyl sulfate–polyacrylamide gel electrophoresis (SDS-PAGE). The concentration of the protein of interest was determined using the BCA Protein Assay Kit.

*Sequence analysis of Pl*GH3.

The nucleotide sequence and amino acid sequence were analyzed using the online blast services of NCBI and Uniprot, respectively. Proteins with high similarity to *Pl*GH3 and clear crystal structures were retrieved using the advanced search function of PDB website. Protein sequences were analyzed using MEGA11, and the multiple sequence alignment diagrams were visualized using the ESPript 3.0.

### Enzymatic activities and characterization of *Pl*GH3

Enzymatic activity assays of purified recombinant *Pl*GH3 were performed using generic aryl-glycosides. The reaction mixture was composed of 2 mM aryl-glycoside in 500 µL of 50 mM sodium phosphate buffer with a certain amount of purified recombinant *Pl*GH3; the enzyme’s working concentration was pre-tested and adjusted to 0.02 mg/mL. After 5 min for reaction, 500 µL of 1 mM Na_2_CO_3_ was added to terminate the reaction. The amount of released chromogenic *p*-nitrophenol or *o*-nitrophenol was immediately examined by the absorbance at 405 nm (Larsbrink et al. 2014). One unit (U) of activity of purified recombinant *Pl*GH3 as glycosidase was referred to as the amount of enzyme required to generate 1 µmol of *p*NP (*o*NP). The effect of pH on the enzymatic activity of *Pl*GH3 was independently performed at 37 °C using 2.0 mM of *p*NP in various buffers: pH 5.0 sodium acetate buffer, pH 6.0 to 8.0 sodium phosphate buffer, and pH 9.0 glycine-sodium hydroxide buffer. The pH stability of purified *Pl*GH3 was investigated by calculating the residual enzymatic activity after incubation in each buffer for 24 h at 4 °C. The effect of temperature on *Pl*GH3 activity was tested by determining the remaining enzymatic activity after incubation for 5 min at different temperatures. The thermostability was measured by analyzing the residual activity after incubation of *Pl*GH3 (0.2 mg/mL) in 50 mM of pH 8.0 sodium phosphate buffer. The effects of metal ions and chemical reagents on *Pl*GH3 activity were determined by maintaining a purified enzyme for 1 h at 30 °C in the presence of 1 or 10 mM of various ions and reagents. Kinetic parameters were performed using freshly purified recombinant enzymes using Ro and *p*NP-*β*-D-Glc at appropriate concentrations. One unit of enzymatic activity was defined as the amount of protein required to produce 1 µmol of ZR1 or *p*NP per minute.

### Biotransformation of TSPJ by *Pl*GH3 and preparation of rare natural oleanane-type saponins

TSPJ was used as the substrate for converting to desired rare saponins by *Pl*GH3 in 50 mM of pH 8.0 sodium phosphate buffer at 37 °C for 2 h. The biotransformation reaction was terminated by adding an equal volume of water-saturated *n*-butanol. After extraction twice, the *n*-butanol fraction was dried with nitrogen and then resuspended with methanol to further analysis by HPLC. To prepare biotransformation products, the enzymatic reaction was conducted with a catalyst loading of lyophilized cell-free extract of *Pl*GH3 in a 1:5 mass ratios to TSPJ in 300 mL of pH 8.0 sodium phosphate buffer for 6 h at 37 °C. The reaction mixture was then extracted twice with equal amounts of water-saturated n-butanol. The resultant *n*-butanol layer was combined and concentrated in a rotary evaporator at 55 °C. The converted products were dissolved in methanol and further separated through a semi-preparative liquid phase system equipped with a Shin-pack PRC-OZ-H column (20.0 mm × 25 cm, 5 µm). The chromatographic separation was achieved by detection at 203 nm in an isocratic elution of 52% acetonitrile with 0.05% formic acid as the mobile phase. The fractions containing target compounds were collected and concentrated in a rotary evaporator at 55 °C, respectively.

### Analysis and identification of products

HPLC analysis was obtained by Agilent 1260 Infinity HPLC system (Agilent Technology, Santa Clara, CA, USA) equipped with a ZORBAX SB-C18 column (4.6 mm*250 mm, 5 µm) at 35 °C. Acetonitrile (solvent A) and 0.05% phosphate (solvent B) were used as the mobile phases. Gradient elution was performed by the followings: solvent A and solvent B from 23:77 to 40:60 (*v:v*) for 20 min, 40:60 to 75:25 for 10 min, 75:25 to 90:10 for 2 min, and keeping it until 45 min. The flow rate of the mobile phase was 1.0 mL/min, and it was monitored at 203 nm. MS analysis was obtained by Q-TOF 5600^+^ mass spectrometer provided with turbo V sources (AB sciex, USA). The operating parameters of Q-TOF–MS were set as below: full-scan data acquisition from m/z 100 to 1500 in negative mode, ion spray voltage (− 4.5 kV), and collision energy (− 35 eV).

The products prepared by the semi-preparative liquid phase were dried and determined by TLC and HPLC. The samples were then taken and dissolved in pyridine-d5 for NMR analysis.

### Methods for molecular docking experiments

To study the region-specificity of *Pl*GH3 in hydrolyzing ginsenoside Ro, online homology modeling was performed using the Swiss-Model online tool. After optimizing the modeling structure, the open-source Pymol 2.6.0 was used for processing to obtain an appropriate receptor file. The ligand file was generated in PDB format after the 3D chemical structure was plotted using MarvinSketch 23.8. The receptor and ligand files were preprocessed using AutoDockTools 1.5.7. Molecular docking was performed using Autodock Vina 1.2.3. The X, Y, and Z direction grid points were set to 62 × 78 × 62 with a grid spacing of 0.375 Å, and the spatial coordinates were set to 56, 8, and 36. The docking results were processed and analyzed using Pymol and Free Maestro under academic licenses.

## Results

### Cloning, expression, and purification of recombinant glycosidase *Pl*GH3

A full-length coding sequence of 2280 bp encoding 759 amino acids was cloned from *P. lactis* by PCR. The PCR product was sequenced and named *Pl*GH3 (GenBank accession number OR801326). *E. coli* BL21 (DE3) containing recombinant plasmid* p*ET28a-*Pl*GH3 was used for recombinant protein expression of *Pl*GH3. The target protein was purified using Ni–NTA resin, and a band of approximately 90 kDa was observed in SDS-PAGE gel electrophoresis (Figure S2), which is consistent with the predicted molecular weight of 89.5 kDa. The concentration of purified *Pl*GH3 was determined to be 12.31 mg/mL.

### Sequence analysis of *Pl*GH3

As shown in Figure S3, the amino acid sequences were attributed to the glycoside hydrolase family 3 C-terminal domain-containing protein from *Paenibacillus* strains. Among them, the protein sequence of *Pl*GH3 has 99.74% amino acids identities with the one from *Paenibacillus lactis* 1574 (WP_210095648.1). The protein sequence of *Pl*GH3 was then aligned with some associated glycosidase sequences from CAZy and PDB database through the ClustaIW algorithm to construct a maximum likelihood tree. The comparison results of evolutionary trees showed that *Pl*GH3 was closely related to BglB (BAA36161.1) (Hashimoto et al. 1998), BglY (AAX35883.1) (Shipkowski and Brenchley 2005), and BglQM (AFS34656.1) (Cui et al. 2011, 2013), with similarities of 70.57%, 69.21%, and 66.26%, respectively (Figure S4).

**Fig. 2:**
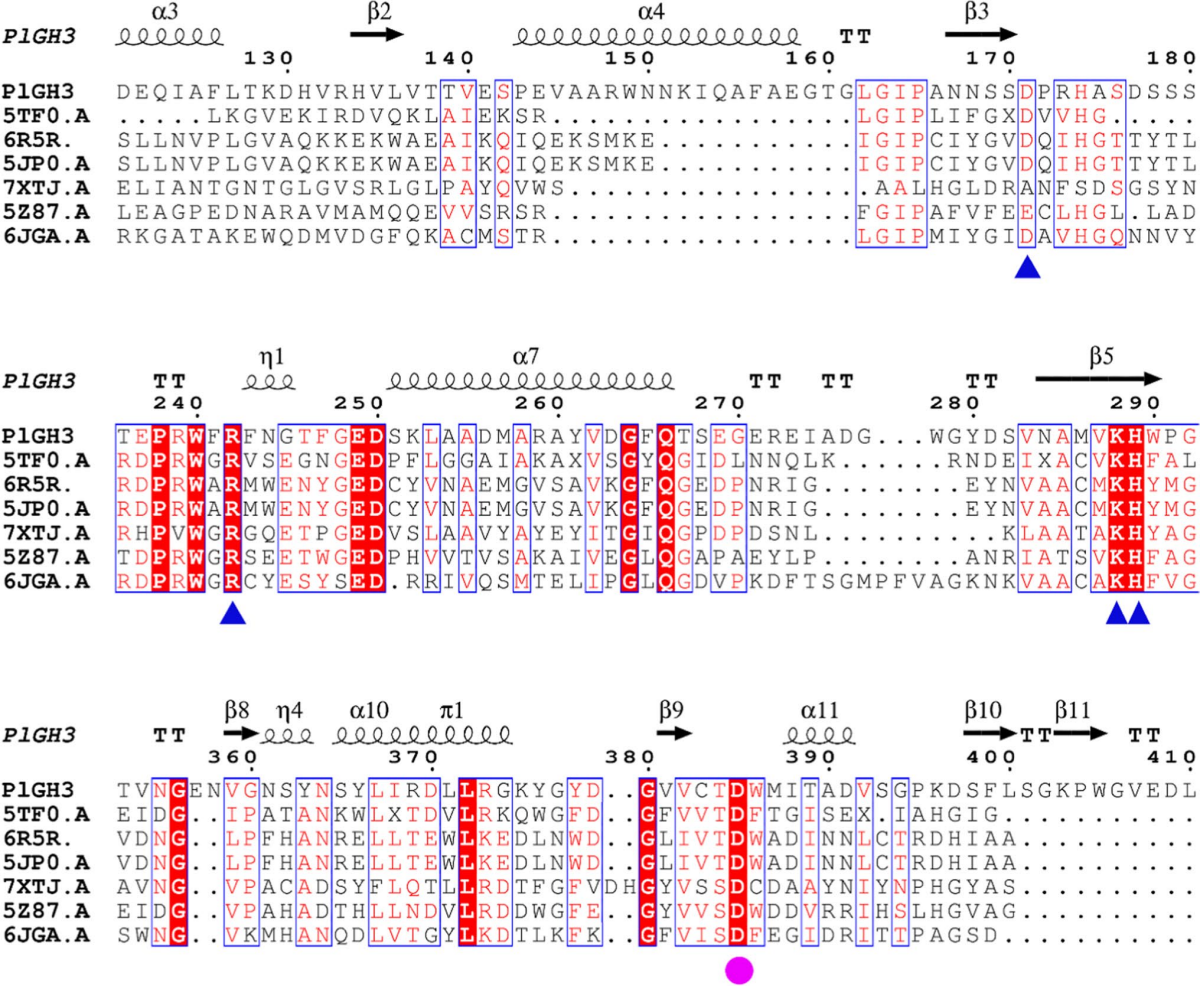
The comparison map was generated by ESPript/ENDscript online tool. The catalytic nucleophile was marked by violet circle. Amino acid residues in the active pocket that form hydrogen bonds with glycosides were marked by blue triangle notations. The original image was displayed in supplementary materials (Figure S5)

The online tool also provides reference templates for homology modeling, all of which are members of the GH3 family. BoGH3B, AnBX, BglX, EmGH1, HvExol, and a glycosyl hydrolase family 3 N-terminal domain protein from *Bacteroides intestinalis* with high similarity were selected for multiple sequence alignment analysis. Their ID in the PDB database were provided in Table S1. These enzymes all have explicit three-dimensional modeling, substrate docking complexes, and characterization studies. Moreover, the details of their active pockets exhibit a high degree of similarity to *Pl*GH3. Therefore, they can be used as references for comparing the hydrolytic mechanisms of the new enzyme. The alignment diagram was generated using ESPript/ENDscript online tool (Fig. 2 and Figure S5). The aspartic acid residues indicated by the purple circles in the figure are crucial amino acids for enzymes in this family, and their positions are highly conserved. On the other hand, the conserved aspartic acid, arginine, lysine, and histidine residues indicated by the blue triangle symbols suggest potential hydrogen bond interactions with substrates in subsequent molecular docking results.

### Enzymatic activities and characterization of *Pl*GH3

The biochemical properties of glycoside hydrolases *Pl*GH3 were characterized in detail by catalyzing aryl glycosides to establish their substrate specificities. The maximum activity sample was marked as 100%. The results shown in Table 1 indicated that *Pl*GH3 has significant activity on *p*NP-*β*-D-Glc, *o*NP-*β*-D-Glc, *p*NP-*β*-D-Gal, and *o*NP-*β*-D-Gal, but no catalytic activity for *p*NP-*α*-D-Glc. Furthermore, the enzyme displays weak activity towards *p*NP-*β*-D-Xyl.

**Table 1:** The hydrolysis activity and substrate specificities of *PlGH3* were measured by catalysis of aryl glycosides; the conversion rate of each reaction was measured under the same reaction conditions. The sample with maximum activity was mark as 100%

Substrate	Relative activity ± SD (%)
*p*-Nitrophenyl-*β*-_D_-glucopyranoside	100 ± 2.5
*o*-Nitrophenyl-*β*-_D_-glucopyranoside	78.3 ± 1.0
*p*-Nitrophenyl-*β*-_D_-galactopyranoside	72.4 ± 1.1
*o*-Nitrophenyl-*β*-_D_-galactopyranoside	84.9 ± 0.6
*p*-Nitrophenyl-*β*-_D_-xylopyranoside	1.24 ± 0.1
*p*-Nitrophenyl-*α*-_D_-glucopyranoside	0

**Fig. 3:**
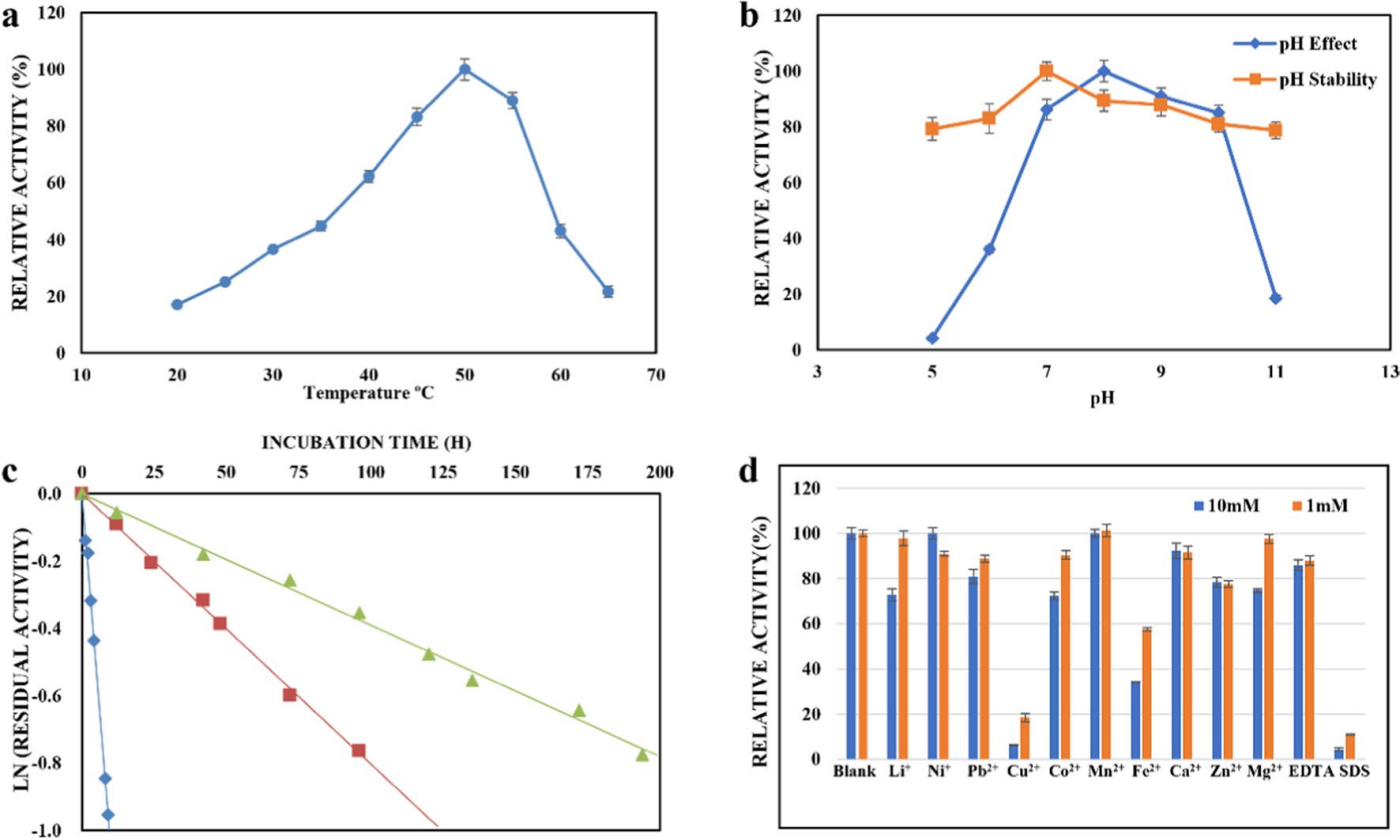
Enzymatic activities and properties of *Pl*GH3: **a** the optimal temperature for *Pl*GH3; **b** the optimal pH and the pH stability of *Pl*GH3;** c** the thermostability of *Pl*GH3; **d** the effects of metal ions and chemical reagents. Data are presented as the mean ± SD from three independent experiments

The optimal pH for *Pl*GH3 was measured to be 8.0. The enzyme showed over 80% of the maximum activity between pH 7.0 to 10.0 (Fig. 3a). However, a significant decrease in enzyme activity was observed as pH approached 6. The enzyme showed good stability by being treated at different pH levels for over 12 h at 4 °C (Fig. 3b). The optimal temperature for *Pl*GH3 was 50 °C, and the reactivity was above 80% at 45 °C or 55 °C. Reactivity only showed less than 50% at 25 °C and 35 °C. The half-lives of *Pl*GH3 at 0.2 mg/mL were 163.85, 79.88, and 6.04 h at 30 °C, 40°C, and 50 °C, respectively (Fig. 3c). Since the presence of metal ions or some chemical reagents affects the structure and function of proteins, different metal ions and some of the reagents common in molecular biology were determined (Fig. 3d). The results showed that the recombinant *Pl*GH3 activities were inhibited significantly on the addition of Cu^2+^, Fe^2+^, and sodium dodecyl sulfate (SDS) at a concentration of 1 mM. EDTA had no significant effect on enzyme activity. Meanwhile, no significant activation was observed on metal ions tested.

The kinetic constant of *Pl*GH3 was defined by the substrate *p*NP-*β*-D-Glc and Ro. The results of *K*_m_, *k*_cat_, and *k*_cat_/*K*_m_ were 22.76 ± 1.33 µM, 46.18 s^−1^, and 2.03 s^−1^·µM^−1^ when *p*NP-*β*-D-Glc as substrate, respectively. It was previously clarified through preliminary experiments that C-IVa, Ro, RT1, and C-IV all share the same C-28 glycolipid bond and can be hydrolyzed by *Pl*GH3 to produce responding conversion products. Given that Ro is a major component in saponin extracts and is relatively easy to obtain, it was chosen as the substrate to study the kinetics of *Pl*GH3. The results of *K*_m_, *k*_cat_, and *k*_cat_/*K*_m_ were 79.59 ± 3.42 µM, 18.52 ± 0.28 s^−1^, and 0.23 s^−1^·µM^−1^ when Ro as a substrate (Figure S6).

### Biotransformation of TSPJ by *Pl*GH3

**Fig. 4:**
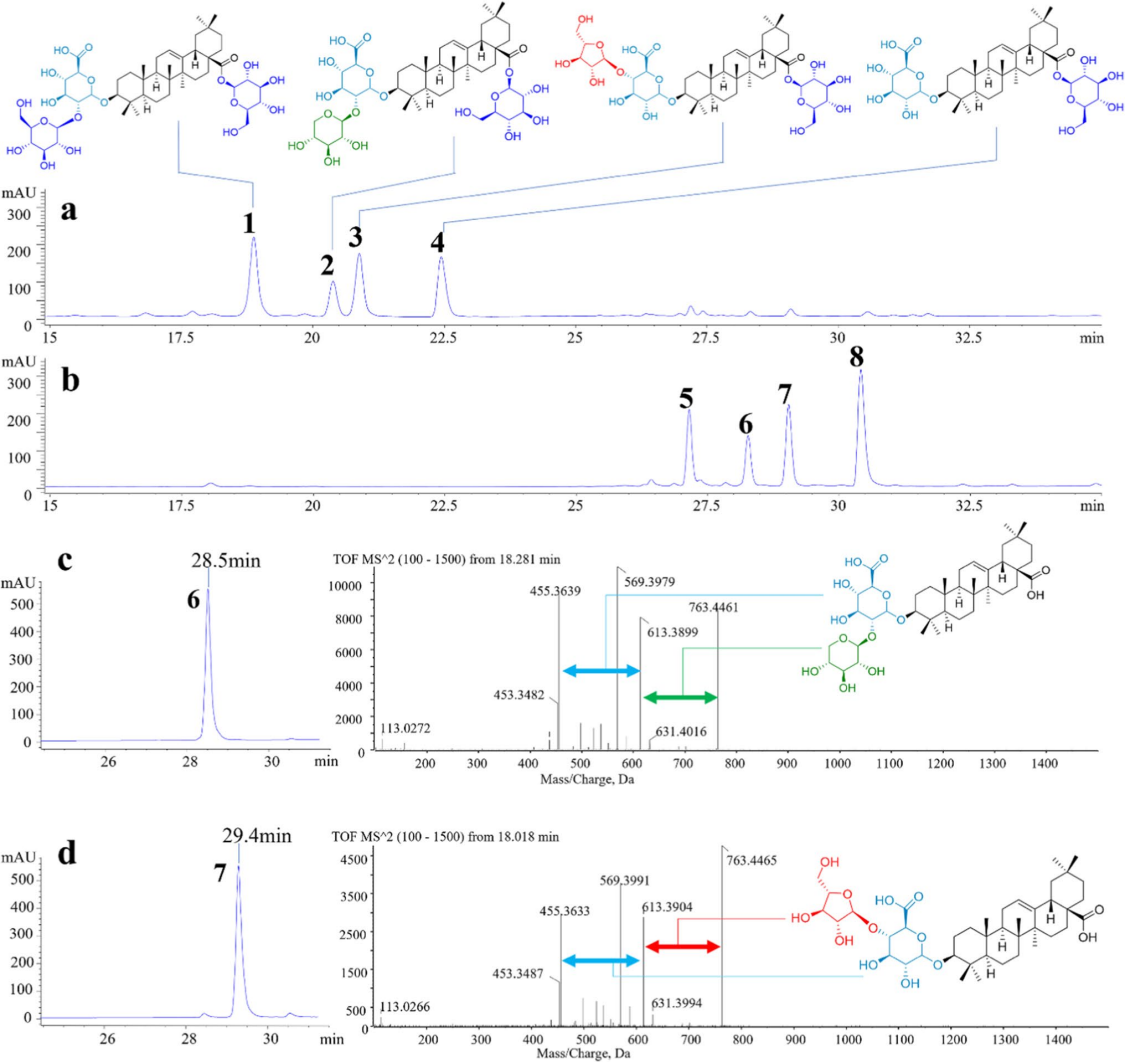
HPLC and MS analysis results of transformation products. **a** HPLC showed the compounds contained in TSPJ include Ro (1), RT1 (2), C-VI (3), and C-IVa (4). Glucose group is represented in blue, and xylose group is represented in green. Arabinfuran group is represented in red, and glucuronic acid group is represented in light blue. **b** HPLC results after the transformation. Four transformation products were determined. **c** and** d** Compounds 6 and 7 were purified by semi-preparative liquid phase separation, with secondary ions by mass spectrometry and inferred fracture patterns

**Fig. 5:**
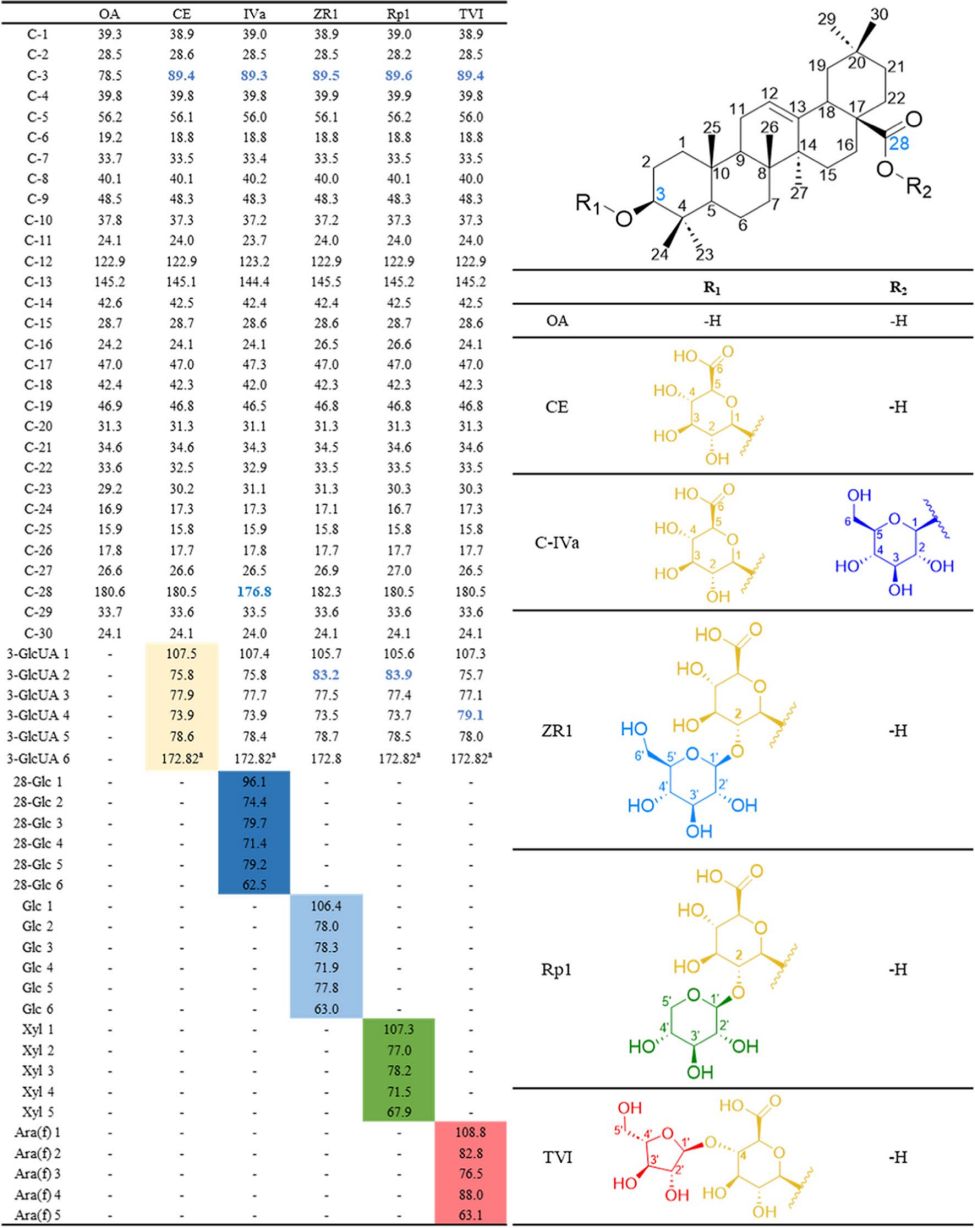
^13^C NMR chemical shifts (δ, ppm) of oleanane acid and oleanane-type saponins in pyridine-d5. **a** Due to its special chemical environment, C-6 on glucuronic acid may not be able to repeat stably in 13C-NMR. The values here are for prediction and was only detected in the ZR1 sample. GlcUA, *β*-D-glucuronic acid; Glc, *β*-D-glucopyranosyl; Xyl,* β*-D-xylopyranosyl; Ara(f),* α*-L-arabinofuranosyl

The HPLC analysis results of TSPJ are shown in Fig. 4a and b, and it turns out that no oleanolic acid was produced in the transformation products. Compounds 1, 2, 3, 4, 5, and 8 were confirmed by standard comparison. Under the treatment of an unpublished xylose hydrolase in our laboratory, Rt1 was converted to C-IVa; the sample without Rt1 was then obtained (Figure S7), which was subsequently treated with *Pl*GH3. It was inferred from the comparison of HPLC results that Rp1 (compound 6) and TVI (compound 7) were the products of RT1 and C-VI after deesterification at C-28 site, respectively. Rp1 and TVI are rare saponins in natural materials; the standards are difficult to obtain. Hence, the reaction system was expanded with 0.5 g of TSPJ treated by *Pl*GH3. The purity compounds (32.1 mg compound 6 and 30 mg compound 7) were successfully prepared after the separation of silica gel column and semi-preparative liquid phase. Meanwhile, the transformation products ZR1 and CE were also obtained. These compounds were subsequently analyzed using mass spectrometry (Fig. 4c, d and Figure S8) and nuclear magnetism ^13^C-NMR (Fig. 5 and Figure S9 ~ S14). Mass spectrometry results show that compounds 6 and 7 both exhibit ionization at *m/z* 619.39, which is expected since xyloside and arabinofuranoside have the same molecular weight. The other fragment ion at *m/z* 455.36 belongs to the product by further the cleavage of glucuronic acid moiety. The ^13^C-NMR spectrum results of the obtained compounds are consistent with those reported in the literature (Yen et al. 2022). The chemical shift of C-3 is 78.5 in the NMR data for oleanolic acid. The chemical shift of C-3 increases to 89.4 ~ 89.6 when it forms a glycosidic bond with glucuronic acid. Compared with the carbon spectrum data of CE and C-IVa, the carbon signals of glucuronic acid groups are *δ*_C_ 107.5, *δ*_C_ 75.8, *δ*_C_ 77.9, *δ*_C_ 73.9, *δ*_C_ 78.6, and *δ*_C_ 172.8, respectively. As shown in the spectrum data of ZR1 (*δ*_C_ 83.2), their chemical shifts increase when these carbons are connected by glycosidic bonds. In contrast, compounds 6 and 7 have different signals of C-2 (*δ*_C_ 83.9) and C-4 (*δ*_C_ 79.1), respectively, which proves the position of glycosidic linkage outside the sugar chain. The characteristic signal of xylose is *δ*_C_ 107.3, *δ*_C_ 77.0, *δ*_C_ 78.2, *δ*_C_ 71.5, and *δ*_C_ 67.9, while that of arabinofuranose is consistent with literature reports at *δ*_C_ 108.8, *δ*_C_ 82.8, *δ*_C_ 76.5, *δ*_C_ 88.0, and *δ*_C_ 63. Therefore, compounds 6 and 7 are identified as Rp1 and TVI, respectively.

### Molecular docking

Subsequently, the structure of *Pl*GH3 was predicted by Swiss-Model online tool under default setting conditions. Templates with high sequence similarity to the *Pl*GH3 sequence were chosen for modeling. Modeling results were ranked based on the Global Model Quality Estimate (GMQE) score. The template 5JP0 exhibited a GMQE of 0.49, and a QMEANDisCo Global value of 0.57 ± 0.05. The obtained results showed a slightly higher score compared to others (Figure S15). The model generated with 5JP0 as the contrast template was used as the docking macromolecular receptor. The template was converted to a suitable PDB file by Pymol and subsequently preprocessed with the receptor using the Autodock Tool. The docking results of *Pl*GH3 with *p*NP-*β*-D-Glc (Figure S16a) were compared to the three-dimensional structure of the GH3 family enzymes, which have been thoroughly characterized and elucidated active pockets with key amino acids (Dai et al. 2018; Kaenying et al. 2023; Larsbrink et al. 2014). Due to the differences between glycosyl esters and glycosyls, the stability of the oxygenated carbo-positive ion-like transition state may be affected when it was formed during glycosylation. Hence, the virtual compound with a structure of perhydrophenanthrene esters has been designed to mimic the molecular structure of oleanane-type saponin (Figure S16b).

**Fig. 6:**
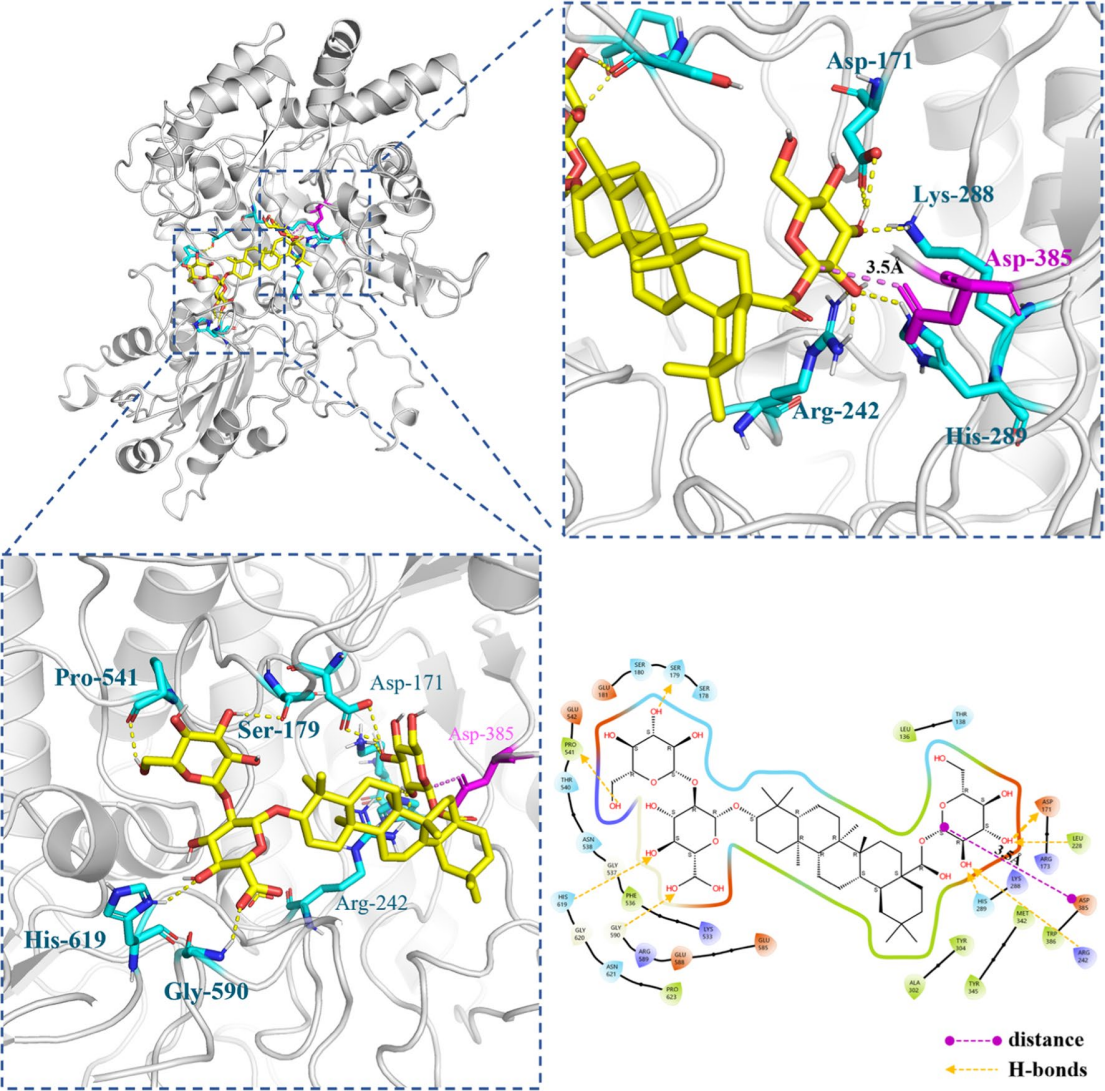
The molecular docking results of ginsenoside Ro and *Pl*GH3. The key amino acid residue Asp385 as a nucleophile is marked as magentas, and the distance from anomeric carbon is shown to be 3.5 Å. The key amino acid residue that creates hydrogen bonding with hydroxyl groups on glucose is labeled in cyan blue

**Fig. 7:**
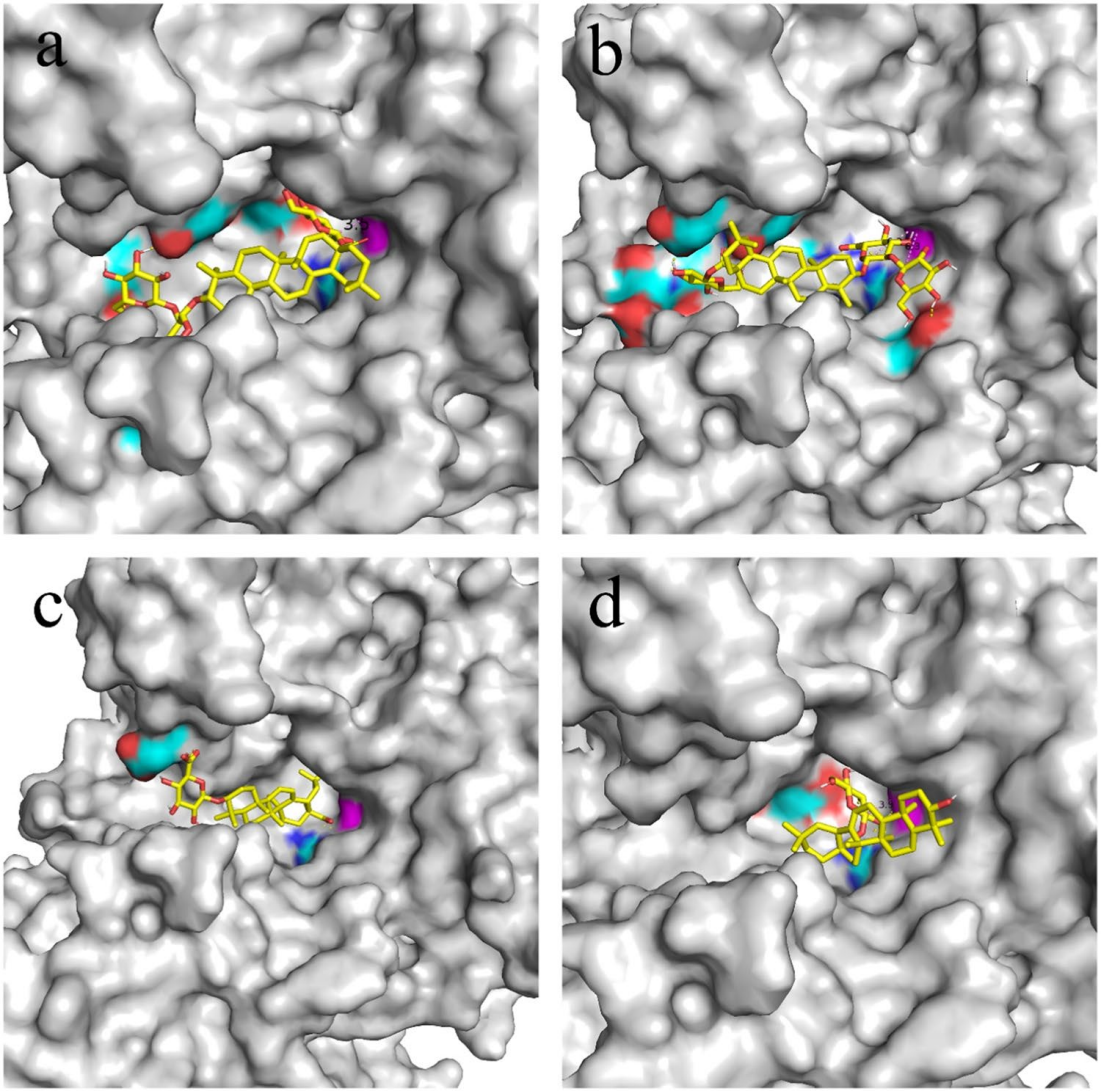
The docking results of different ligands with *Pl*GH3. Magenta color on the surface indicates the key amino acid Asp385. The red region represents the region that can produce hydrogen bonds with the ligand, and the cyan blue indicates the location of the amino acid residue that produces hydrogen bonding. **a** and **b** show Ro entering the active pocket in two opposite postures, respectively. **c** and **d** show the entry of oleanolic acid glycyl ester and calendulin E into the pocket center in the lowest energy state, respectively

Oleanane-type saponin has a certain rigidity, and glycan substitutions occur at both ends of the molecule (C-3 and C-28). The active pocket of *Pl*GH3 is a groove shaped like Taco and is suitable for the entry of molecules such as oleanane-type saponin. The molecular docking results of ginsenoside Ro and *Pl*GH3 are shown in Figs. 6 and 7a. Although Ro can enter the active pocket in two diametrically opposed positions, only the conformation of the C-28 end close to Asp385 obtains the lowest binding energy. Asp171, Lys288, Arg242, and His289 participate in the formation of hydrogen bonds, and nucleophiles attack anomeric carbon on pyran rings as far as 3.5 Å. Notably, the glycan at the other end can form hydrogen bonds with amino acid residues Pro541, Ser179, Gly590, and His619 outside the active pocket, further improving the stability of this bound state. When the end of C-3 is extended into the center of the pocket, the number of hydrogen bonds formed at both ends of Ro is less, and the distances between Asp385 with the anomeric carbons of the glucose group and glucuronic acid group are up to 6.2 Å and 6.3 Å, respectively, which is difficult to make a chemical reaction (Figure S17 and Fig. 7b).

In addition, some structurally similar compounds have also been analyzed as ligands for molecular docking (Figure S18). C-IVa have sugar chains at both ends, and each of which can form hydrogen bonds with amino acid residues. There are two opposite binding conformations, but the binding results in Figure S18a are more favorable with Asp385 at 3.4 Å from the anomeric carbon, less than 4.9 Å from the conformation shown in Figure S18b. The docking results for ZR1 are shown in Figure S18c and S18d, where Figure S18c is the best state for docking evaluation. The docking state of ZR1 shown in Figure S18d has a lower rating, although the glycan chain on the C-3 side can still form hydrogen bonds with the amino acid residues in the active pocket, the distance between the anomeric carbon and Asp385 is increased to 5.1 Å due to the spatial structure of the glycan group, and the hydrolysis reaction is not triggered. There is no glycosylation on the C-28 side of CE, and the lowest energy configuration is shown in Figure S18e, and the glucuronic acid group on the C-3 side produces hydrogen bonding with the Glu181 outside the pocket. The result of oleanolic acid-β-d-glucopyranosyl is as the opposite of those of CE, the state in Figure S18f appears in the docking results, and the molecule can swing due to the lack of anchoring at the C-3 site.

## Discussion

The study of the isolation and preparation of natural products has always attracted a great deal of attention. However. the shallow content of rare natural products in plant materials and lack of sufficient material supply are the key bottlenecks that limiting the pharmacological research. Enzymatic conversion via targeted glycoside hydrolysis is an efficient approach to obtain rare valuable natural products with excellent specific activity. Deglycosylated oleanane-type ginsenosides have important pharmacological applications but are often rare in natural medicinal plants (Wang et al. 2021b). In previous studies, RP1 is considered to have the activity on adjuvant analgesia (Jansakul et al. 1999), and TVI has been proven to have the functions of cytotoxic effects towards three human cancer cell lines (Yen et al. 2022). In addition, ZR1 has shown pharmacological activities on antitumor, neuroprotective, and antioxidant effects (Sayed et al. 2022), and C-IVa has been recently confirmed to alleviate myocardial ischemia/reperfusion injury (Wang et al. 2019). These studies suggest that these rare saponin compounds are worth further investigation and development. Therefore, developing a method for the efficient and environmentally friendly production of these rare saponins is highly meaningful.

*Pl*GH3 can be rapidly and abundantly obtained through an *Escherichia coli* recombinant expression system, which is a crucial requirement for its application. The nucleotide and amino acid sequences of the enzyme were studied, revealing its similarity to enzymes such as BglB, BglY, and BglQM in the Gh3 family. Among them, BglB originates from *Bacillus* sp. GL1 and belongs to the GH3 family. It is noteworthy that this enzyme can hydrolyze *p*NP-*β*-D-Glc but cannot hydrolyze *p*NP-*β*-D-GlcA. BglY, derived from *Paenibacillus* sp., exhibits high activity towards *o*NP-*β*-D-Glc and demonstrates approximately 56% hydrolytic activity towards *p*NP-*β*-D-Glc. On the other hand, BglQM, sourced from *Mucilaginibacter* sp. strain QM49, shares high activity towards *o*NP-*β*-D-Glc similar to BglY, but it lacks hydrolytic activity towards *p*NP-*β*-D-Glc. Importantly, BglQM is known to specifically hydrolyze ginsenosides Re and Rg1, producing ginsenoside (*S*)-Rg2 and (*S*)-Rh1. The performance demonstrated by PlGH3 in terms of its optimal pH range and metal ion independence is comparable to that of the aforementioned enzymes. Enzymatic characterization of *Pl*GH3 confirmed that the optimal reactive temperature was 50 °C and efficient conversion can be achieved in a wide range of pH from neutral to weak alkaline. This differs from most of the GH3 family enzymes that have been reported, and usually the optimal environment for GH3 enzymes is pH 4.0 to 6.5 (Zhou et al. 2017). It is important to emphasize that ester bonds at position C-28 can be hydrolyzed under controlled alkaline conditions (Zhu et al. 2014), but this process requires the use of strong alkaline reagents, maintaining pH 11, and high temperature heating. Under the conditions of this biotransformation (pH 8, 37 °C), the C-28 ester bonds of saponins such as ginsenoside Ro were not significantly degraded. Meanwhile, the glucose esters and glycosides have similar structures and can both be hydrolyzed under appropriate acidic or basic conditions. They can be catalyzed by some broad-spectrum glycoside hydrolases. Snailase was used to hydrolyze ginsenoside Ro (Fan et al. 2021). This common commercial hydrolase can hydrolyze the glucose on the outside of the C-3 position and the glucose ester on the C-28 position at the same time. The conversion products of Ro include C-IVa, ZR1, and CE. This general glycoside hydrolase lacks specificity in hydrolysis regions and is not suitable for the preparation of targeted specific products. Therefore, the biotransformation protocol based on *Pl*GH3 avoids the shortcomings of the chemical method of using strong alkali and high temperature and has higher specificity and specificity than the biotransformation using snailase.

In the characterization study results, the enzyme demonstrated non-dependence on ions. The additional supplementation of ions such as Mg^2+^ or Ca^2+^ did not significantly enhance hydrolytic activity, whereas the addition of Cu^2+^ or Fe^2+^ resulted in a reduction. In the past cases, the inhibitory effect of Cu^2+^ and Fe^2+^ ions on GH3 family enzymes has been widely observed (He et al. 2022). SDS may cause protein denaturation by destroying the hydrophobic interaction of proteins; these inhibitory effects are consistent with expectations (Kaushal et al. 2021).

Due to the lack of standards for the expected products RP1 and TVI, the enzymatic reaction system was scaled up. A biotransformation was conducted using 0.5 g of TSPJ as the substrate. The products, Rp1 (32.1 mg) and TVI (30 mg), were ultimately separated and prepared through semi-preparative liquid chromatography. Furthermore, the products were identified by mass spectrometry and nuclear magnetic resonance (NMR).

The specific hydrolytic activity of *Pl*GH3 to the 28-*O*-*β*-d-glucopyranosyl ester bond of oleanane-type saponins is based on its special protein structure. As expected, *Pl*GH3 have a typical (*β*/*α*) 8-barrel structure of the GH3 family (Figure S15). Many experimental results suggest that the catalytic nucleophile residues of GH3 family enzymes from different species sources are highly conserved (Zhou et al. 2017). The catalytic nucleophile residue is Asp and its position is relatively conserved. However, there is diversity in the amino acid residues of acid–base pairs. In this case, as shown in Fig. 2, Asp385 is considered as the catalytic nucleophile. Amino acid residues such as Asp171, Agr242, and Lys288 were conservative, and it has also been stated that these amino acids are used in active pockets to form hydrogen bonds with pyranoses (Fig. 6). As shown in the molecular docking results, the ligand can only enter the reaction center in a special posture. This limitation makes it difficult for saponins to easily extend the C-3 side into the active pocket. As shown in Figure S17 and S18, the unfavorable binding of Ro and the docking results of some structurally similar saponins all support this judgment. As shown in Figure S17, Ro and C-IVa with both C-28 and C-3 glycosyl substitutions theoretically have two binding modes in two positions, but the mode where the C-28 side binds to the reaction center is the dominant conformation. When ZR1 and CE are docked, the dominant conformations in Figure S18c and S18e, respectively, show that the C-3 sugar chain forms hydrogen bonds on the outside of the active pocket, indicating the contribution of transverse hydrogen bonding to the binding. As shown in the docking result of oleanolic acid *β*-d-glucopyranosyl ester (Figure S18f), the molecule can almost swing due to the lack of hydrogen bond anchoring on the outside, further illustrating the contribution of non-conserved amino acid residues on the outside of the active pocket to the stability of the ligand-acceptor complex. Together, these factors support *Pl*GH3 a regionally selective property for the hydrolysis of Ro.

In conclusion, a GH3 family enzyme *Pl*GH3 was screened and thoroughly characterized with special hydrolytic activity of oleanane-type saponin. Molecular docking models were used to try to explain the possible mechanisms of its specificity. With TSPJ as the substrate, four kinds of rare oleanane-type saponins were successfully prepared by an environmentally friendly and efficient biotransformation method. This is the first time that RP1 and TVI have been obtained by biotransformation method, which provides a material basis for subsequent application research of these compounds. This study will provide a meaningful reference for the preparation of high-value rare compounds by biotransformation in the future.

## Supplementary Information

Below is the link to the electronic supplementary material.

Supplementary file1 (PDF 3687 KB)

## Data Availability

The authors declare that the data supporting the findings of this study are available within the paper and its Supplementary Information files. Should any raw data files be needed in another format, they are available from the corresponding author upon reasonable request.
