# Supplementary material for: Novel glycosidase from Paenibacillus lactis 154 hydrolyzing the 28-O-β-d-glucopyranosyl ester bond of oleanane-type saponins
Source: Appl Microbiol Biotechnol. 2024 Apr 4;108:282. doi: 10.1007/s00253-024-13109-2 (PMC10995091; doi:10.1007/s00253-024-13109-2)
Supplement: Supplementary file 1 [file 253_2024_13109_MOESM1_ESM.pdf]

Supplementary materials:

Novel glycosidase from *Paenibacillus lactis* 154 hydrolyzing the 28-O-β-D-glucopyranosyl ester bond of oleanane-type saponins

Zongzhan Wu,[1,2#] Wenyu Dou,[1,2#] Xiaolin Yang,[1,2] Tengfei Niu,[1,2] Zhuzhen Han[1,2], Li Yang,[1,3] Rufeng Wang,[1,2,3]* and Zhengtao Wang[1,2,3]*

[1] Institute of Chinese Materia Medica, [2] The SATCM Key Laboratory for New Resources and Quality Evaluation of Chinese Medicines, and [3] The MOE Key Laboratory for Standardization of Chinese Medicines, Shanghai University of Traditional Chinese Medicine, Shanghai 201203, P.R. China.

*Corresponding authors. Tel.: +86-21-5132-2495; Fax: +86-21-5132-0840; E-mails: wrffrw0801@ shutcm.edu.cn (R.F. Wang); ztwang@shutcm.edu.cn (Z.T. Wang).

#The authors contributed equally.

*Corresponding authors. Tel.: +86-21-5132-2495; Fax: +86-21-5132-0840; E-mails: wrffrw0801@ shutcm.edu.cn (R.F. Wang); ztwang@shutcm.edu.cn (Z.T. Wang).

Supplementary Table and Figures captions:

Table S1

Protein sequences used for reference and their ID in the PDB database.

Protein	PDB ID
BoGH3B	5JPO
AnBX	7XTJ
BglX	6R5R
EmGH1	5Z87
HvExoI	6JGA
A protein from *Bacteroides intestinalis*	5TF0

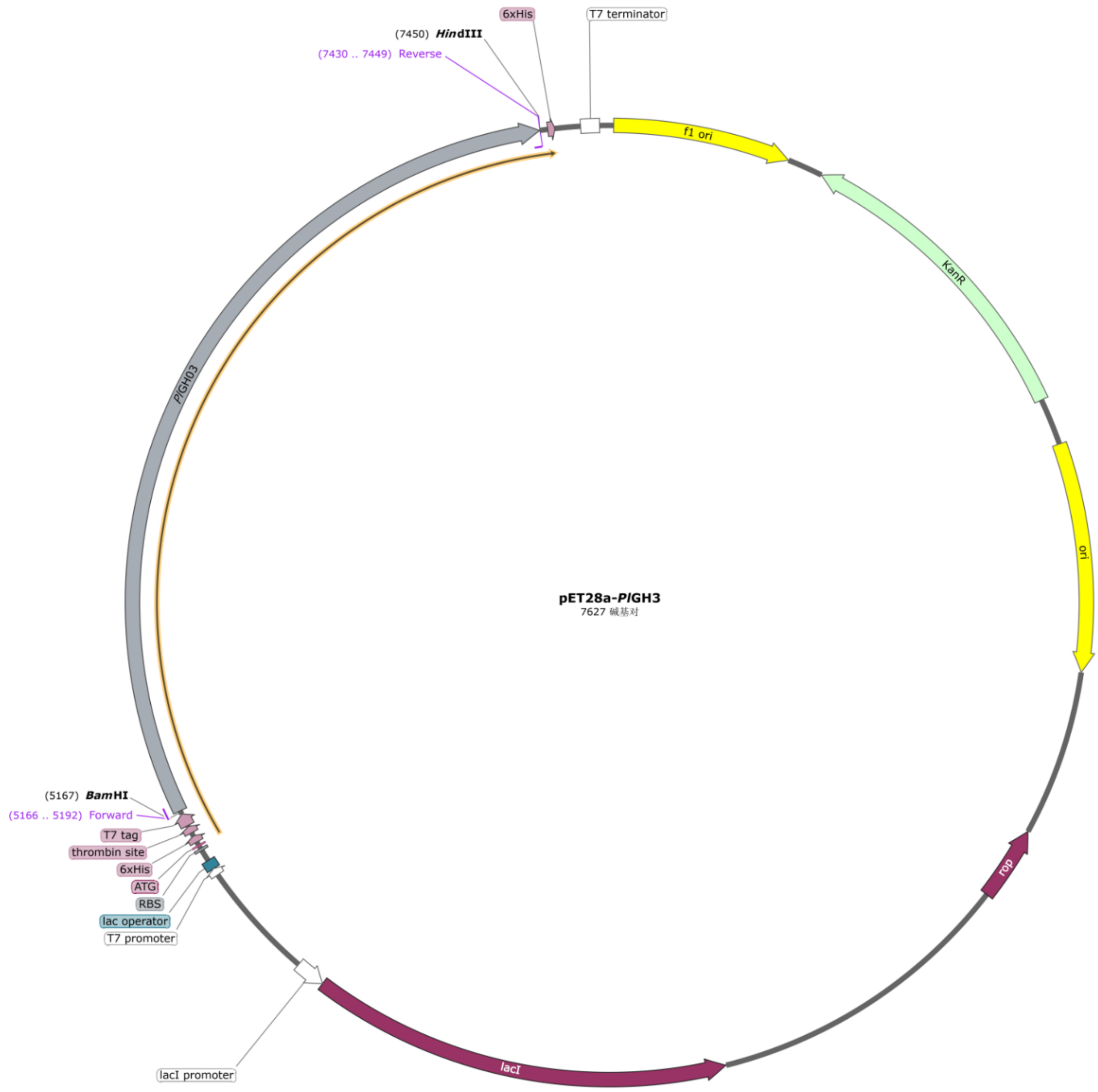

Fig.S1 Schematic diagram of the pET-28a-*Pl*GH3 plasmid.

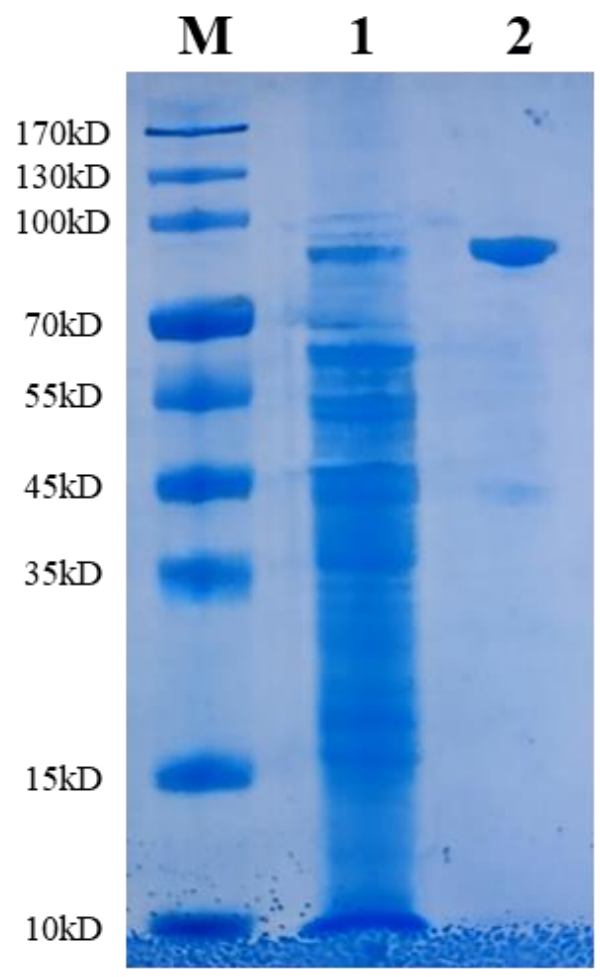

Fig. S2 SDS-PAGE gel with the purification of *Pl*GH3. **M**: molecular weight markers; **1**: Insoluble fraction after sonication; **2**: fraction purified by Ni– NTA affinity chromatography

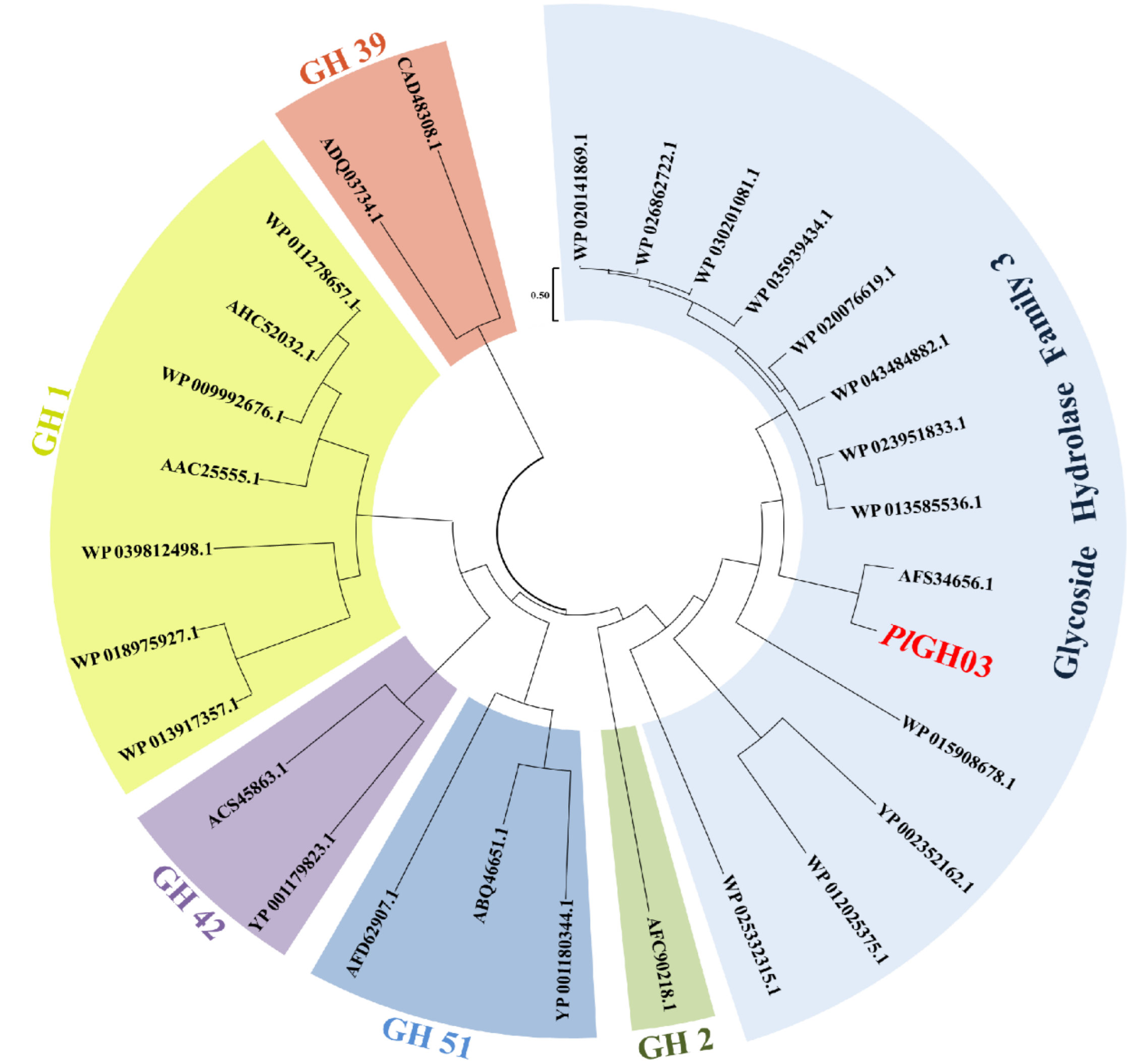

Fig. S3 Several ginsenosidases reported in recent years were compared with *Pl*GH3. Different families of hydrolases are distinguished by different color blocks: light blue for GH3; Green is GH2; Blue for GH51; purple for GH42; yellow for GH1; orange for GH39

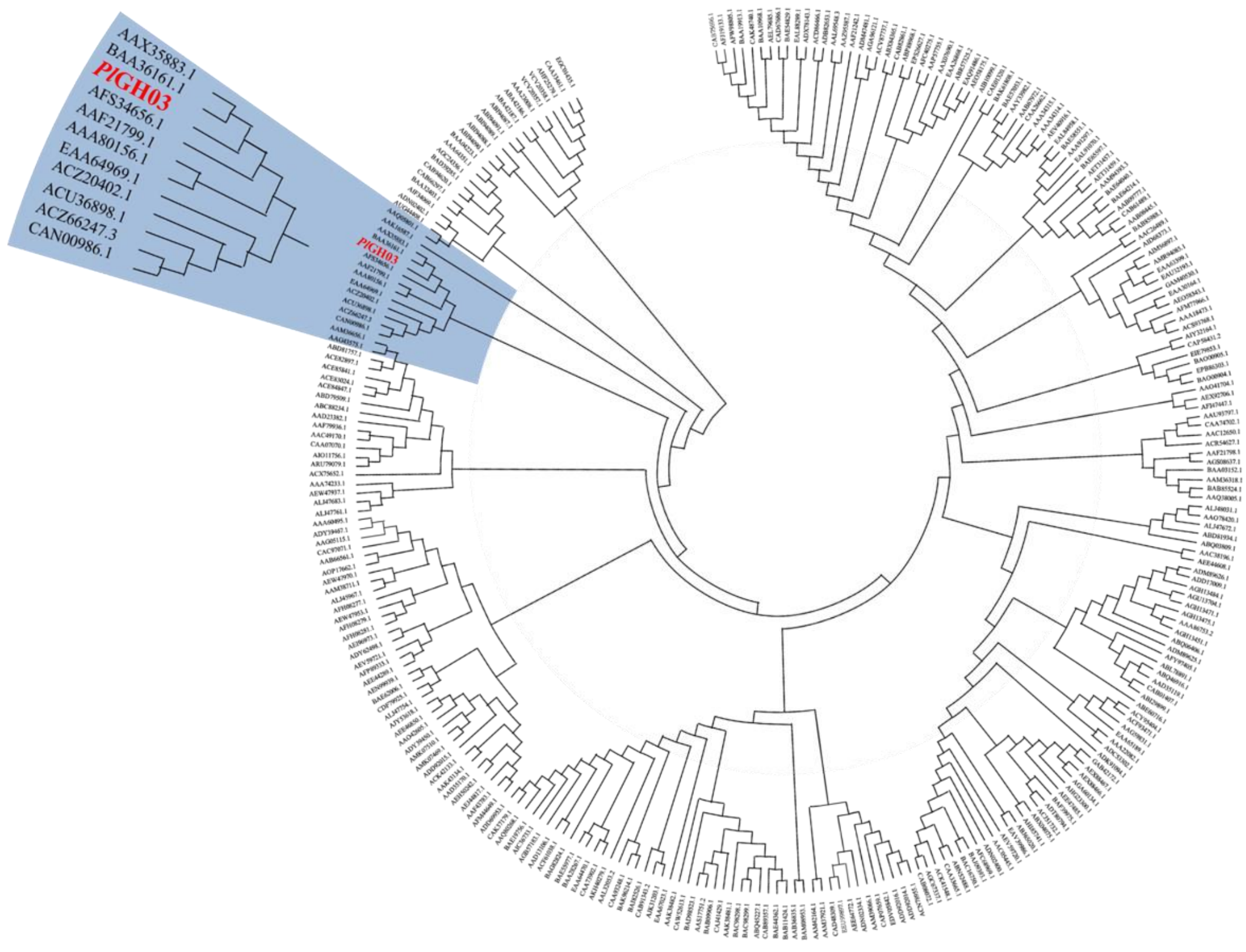

Fig. S4 The evolutionary tree analysis of *Pl*GH3 based on GH3 sequences in CAZy database shows that PlGH3 was closely related to BglB (BAA36161.1), BglY (AAX35883.1) and BglQM (AFS34656.1) with similarity of 70.57%, 69.21% and 66.26%, respectively.

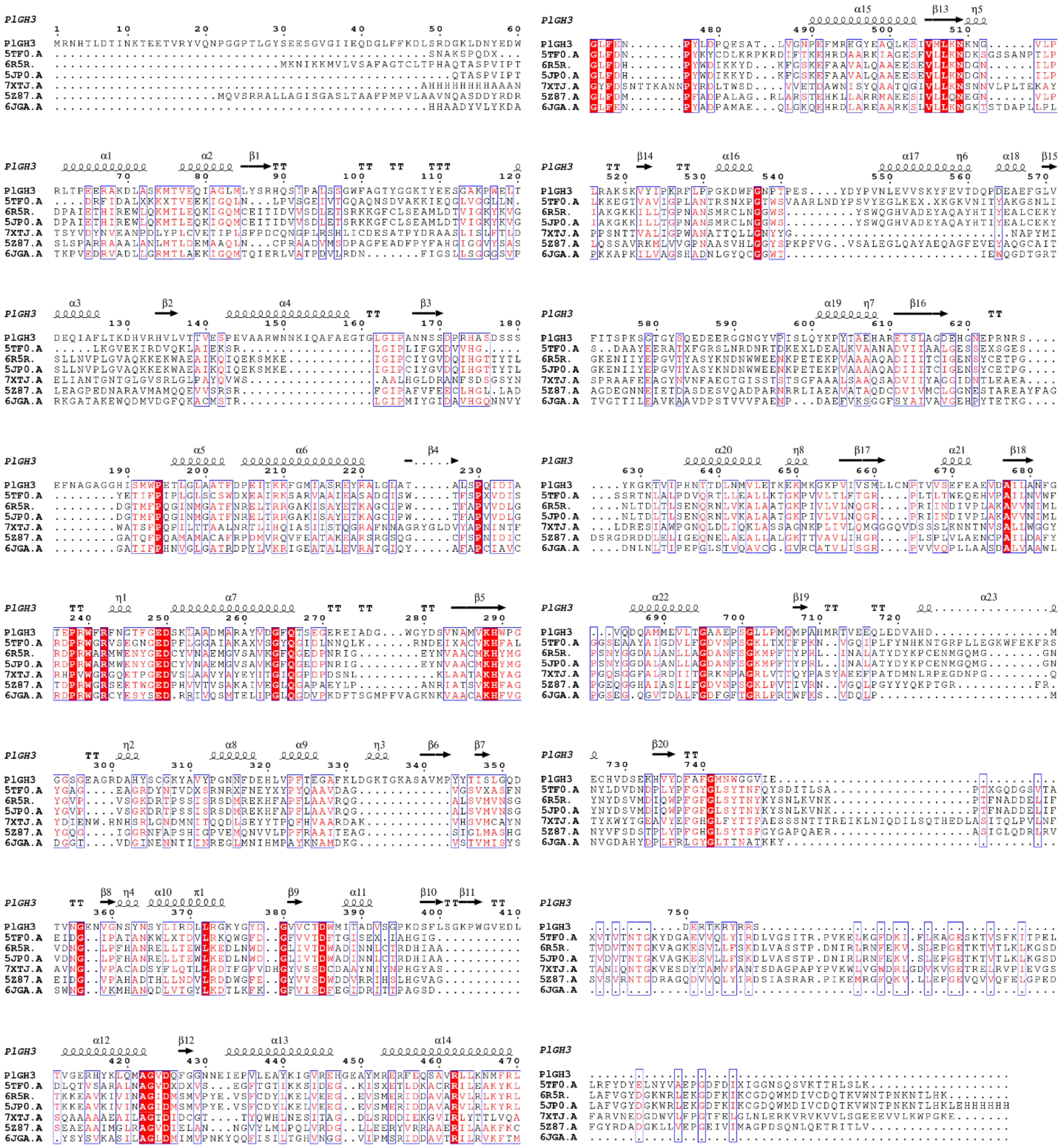

Fig. S5 The multiple sequence alignment of *Pl*GH3 with BoGH3B (PDB ID: 5JPO), AnBX (PDB ID: 7XTJ), BglX (PDB ID: 6R5R), EmGH1 (PDB ID: 5Z87), HvExoI (PDB ID: 6JGA) and a glycosil hydrolase family 3 N-terminal domain protein from Bacteroides intestinalis (PDB ID: 5TF0).

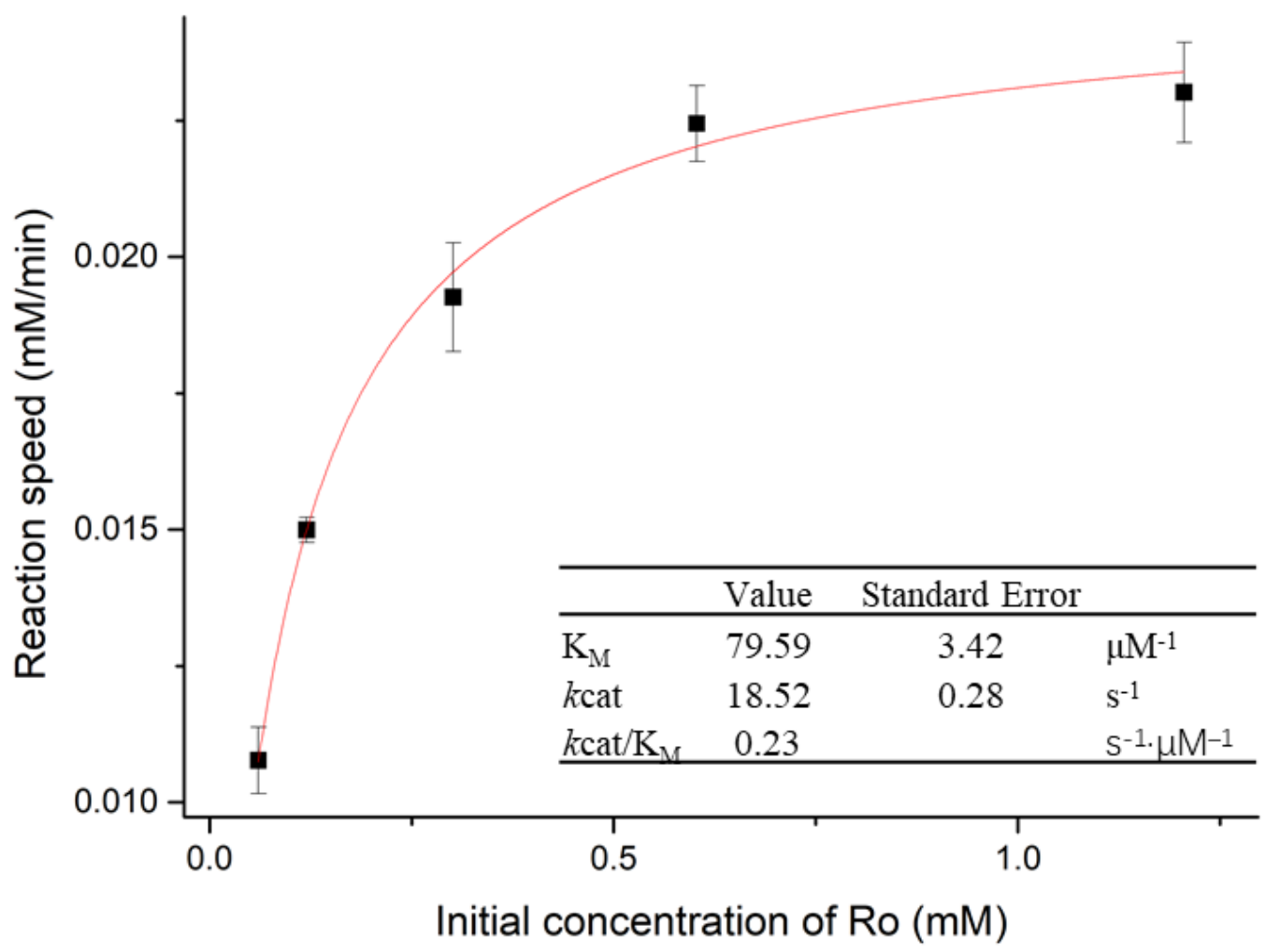

Fig. S6 The kinetic constant of *Pl*GH3 was defined by substrate Ro, The kinetic constants were obtained by curve fitting Michaelis-Menten Equation.

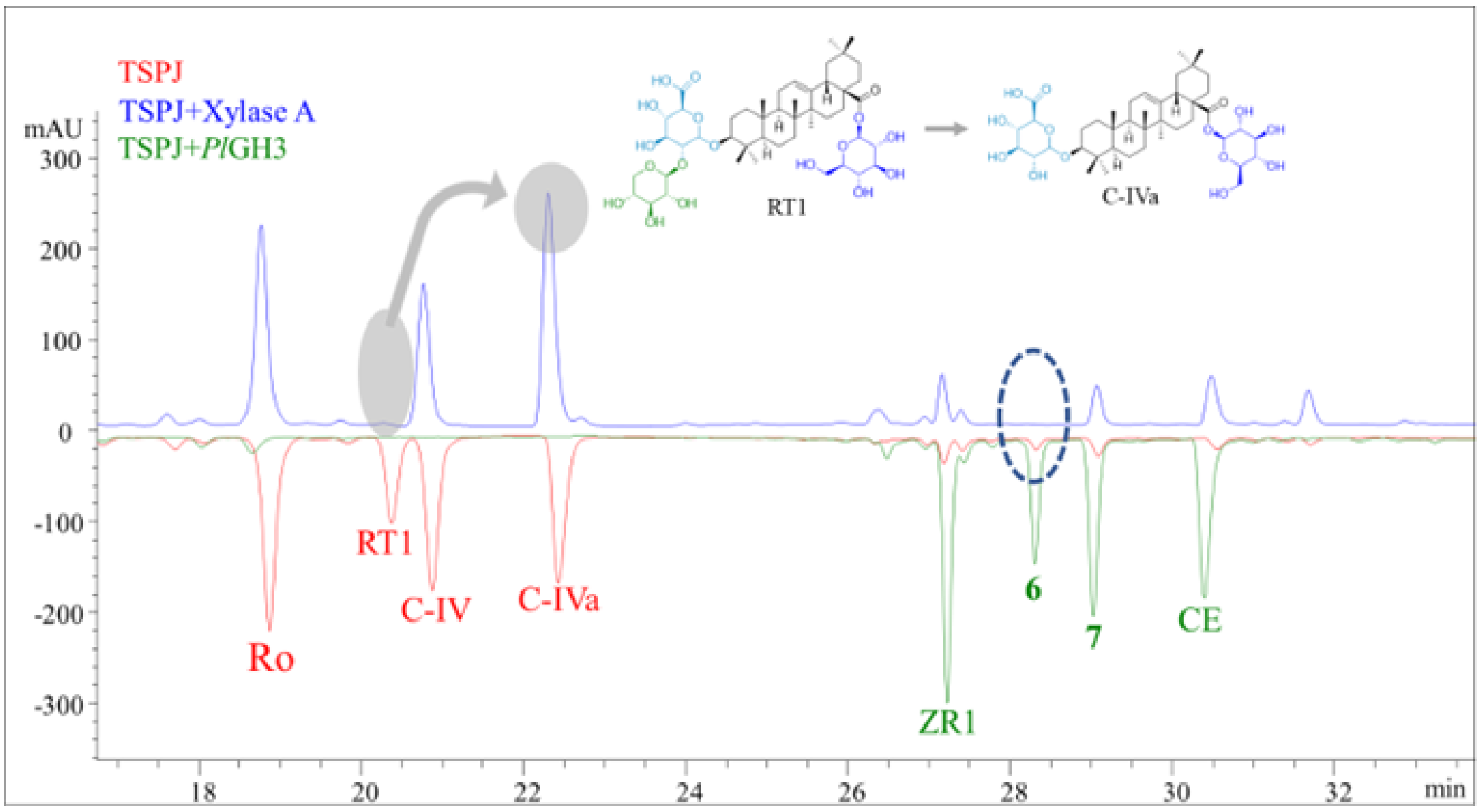

Fig. S7 Under the treatment of an unpublished xylose hydrolase in our laboratory, RT1 was converted to C-IVa, so a sample without Rt1 was obtained.

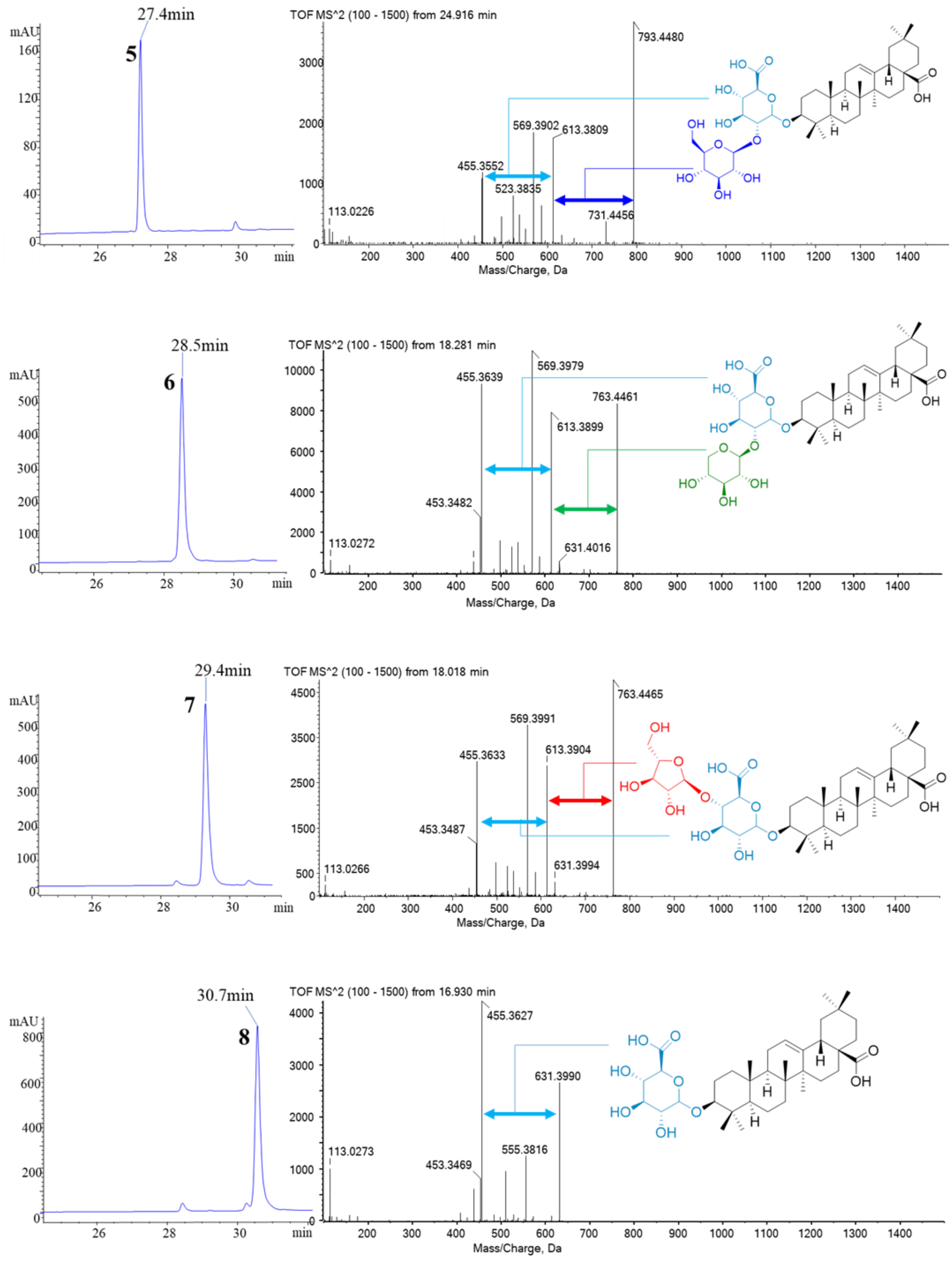

Fig. S8 Mass spectrometric analysis of transformation products.

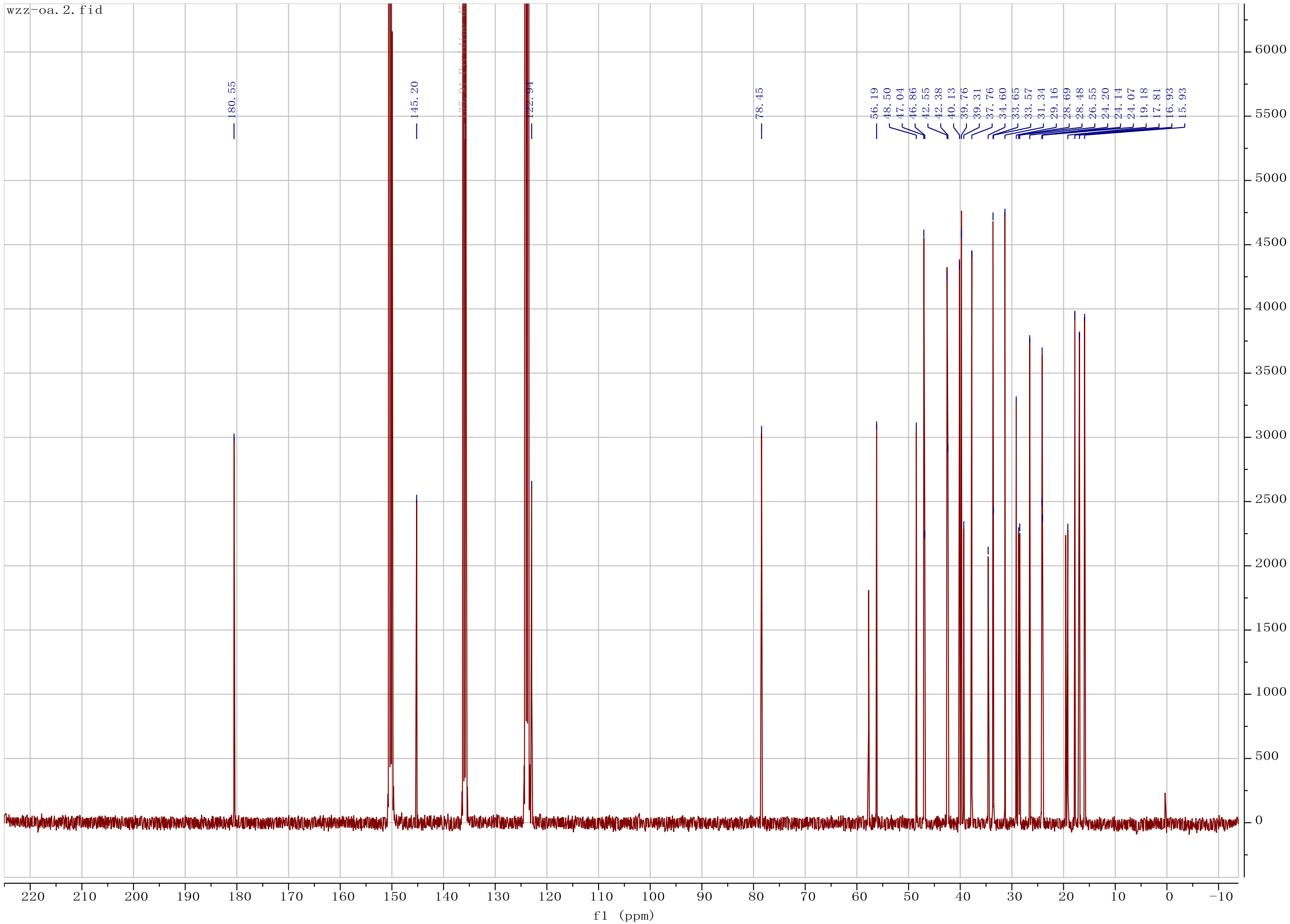

Fig.S9 ^{13}C-NMR results of oleanane

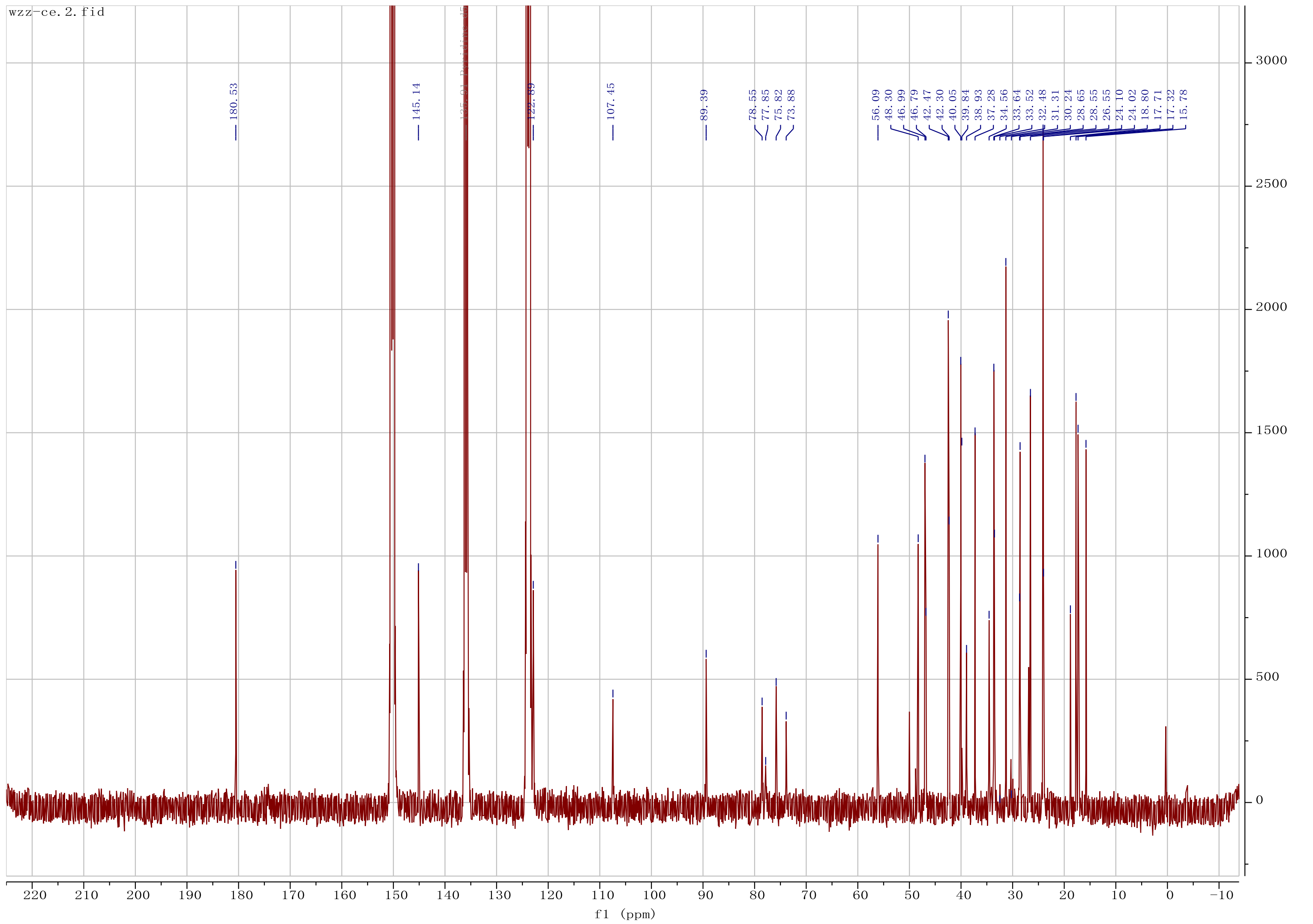

Fig.S10 ^{13}C-NMR results of calenduloside E

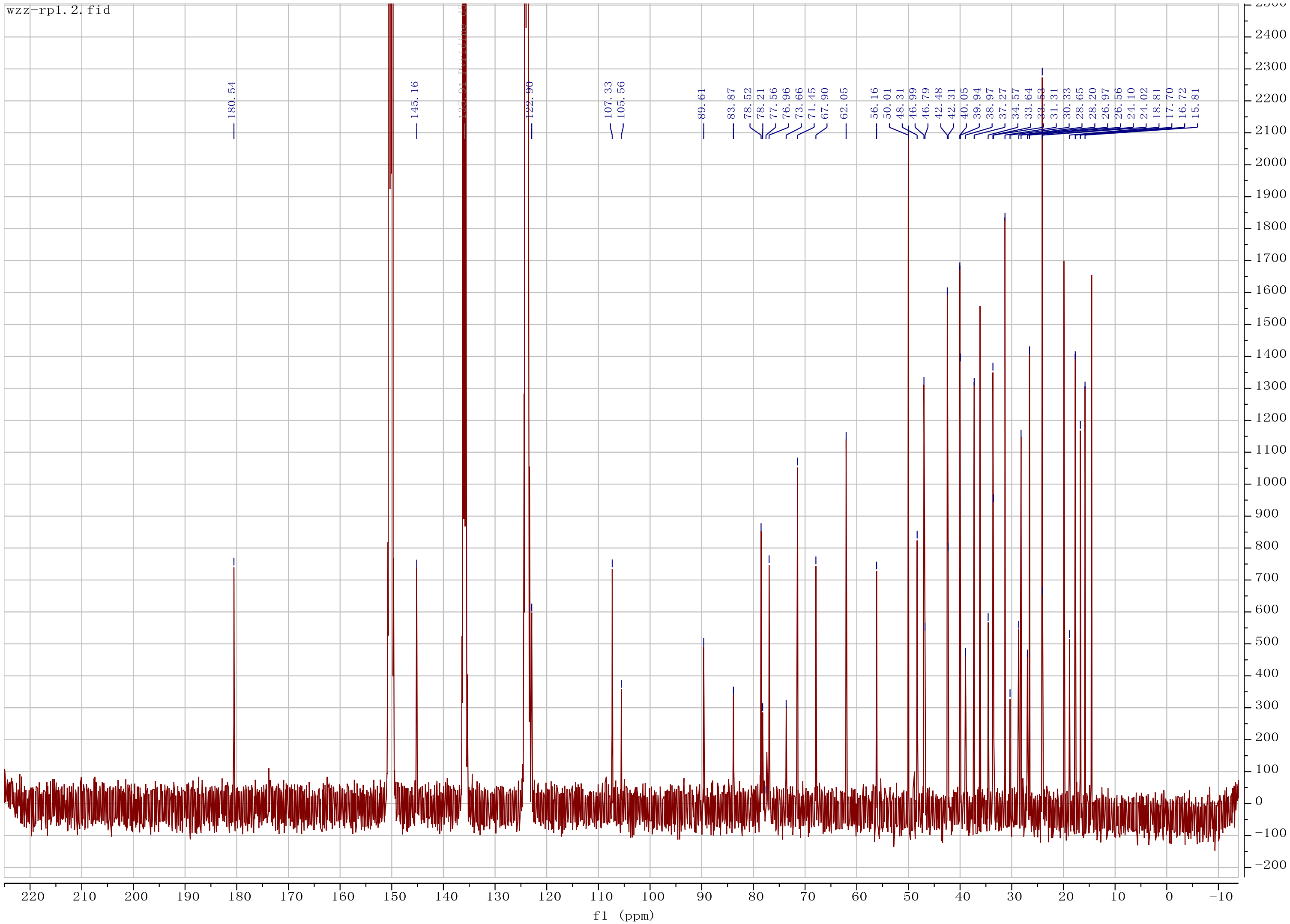

Fig.S11 ^{13}C-NMR results of pseudoginsenoside RP1

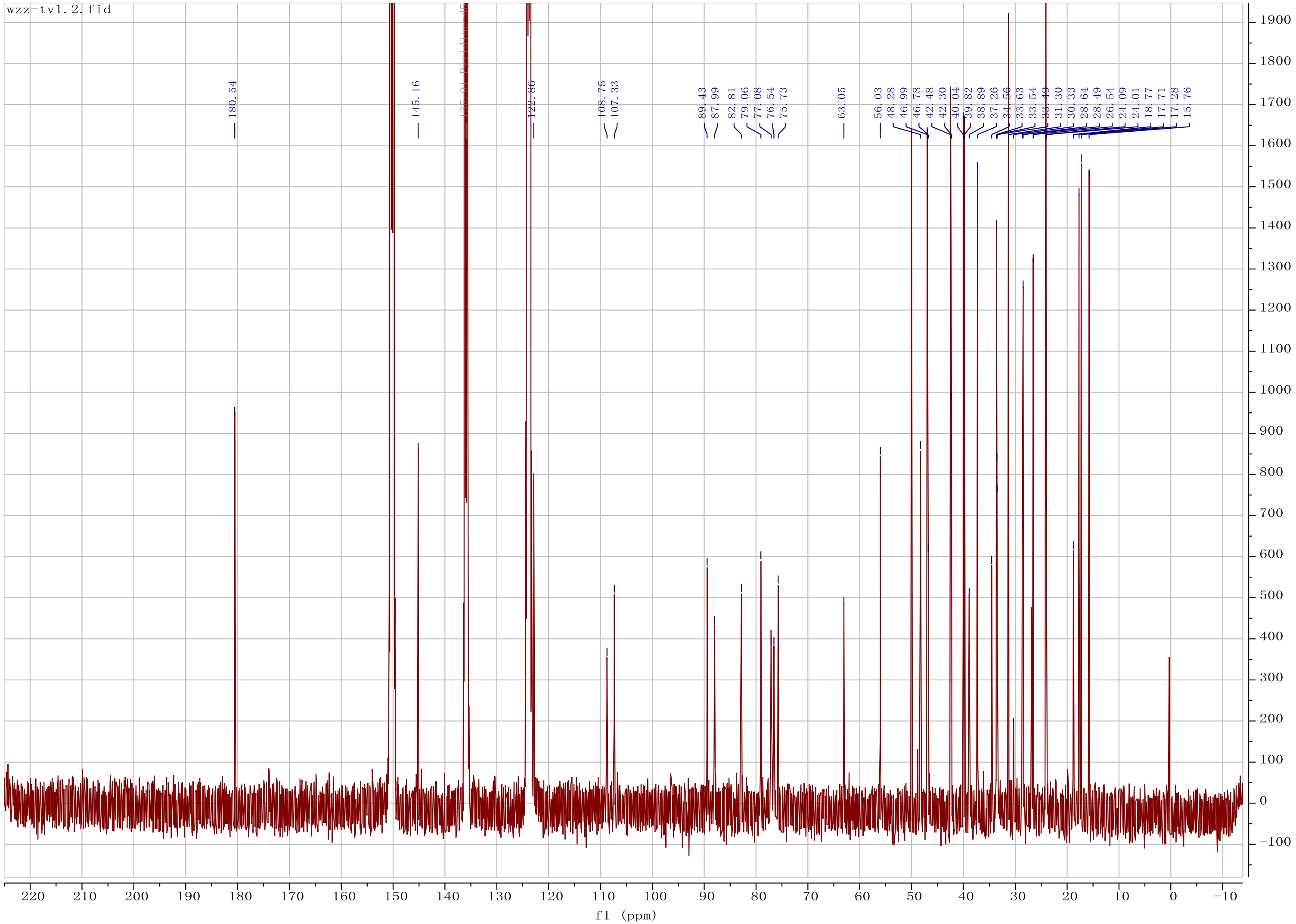

Fig.S12 ^{13}C-NMR results of tarasaponin VI

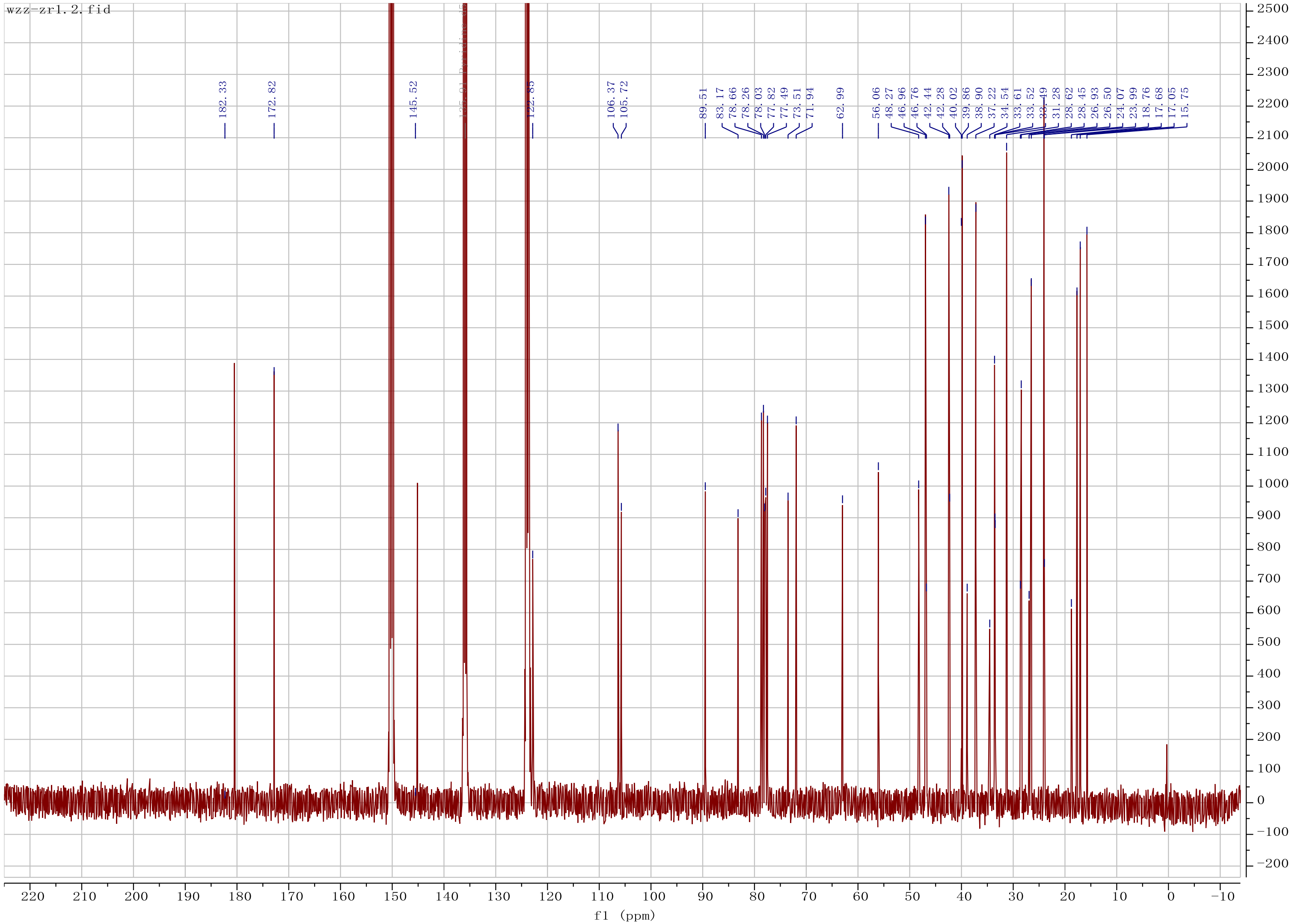

Fig.S13 ^{13}C-NMR results of zingibroside R1

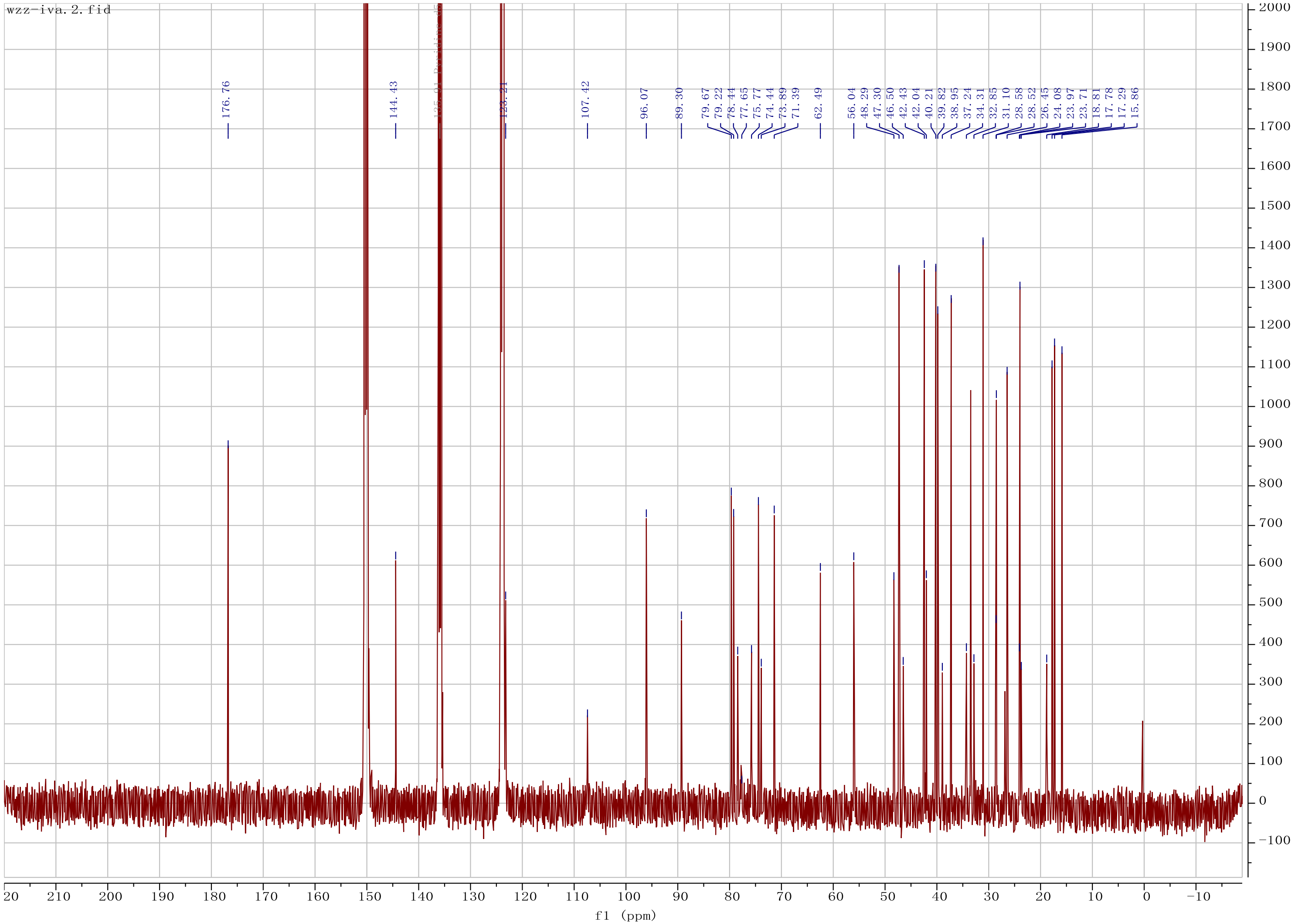

Fig.S14 ^{13}C-NMR results of chikusetusaponin IVa

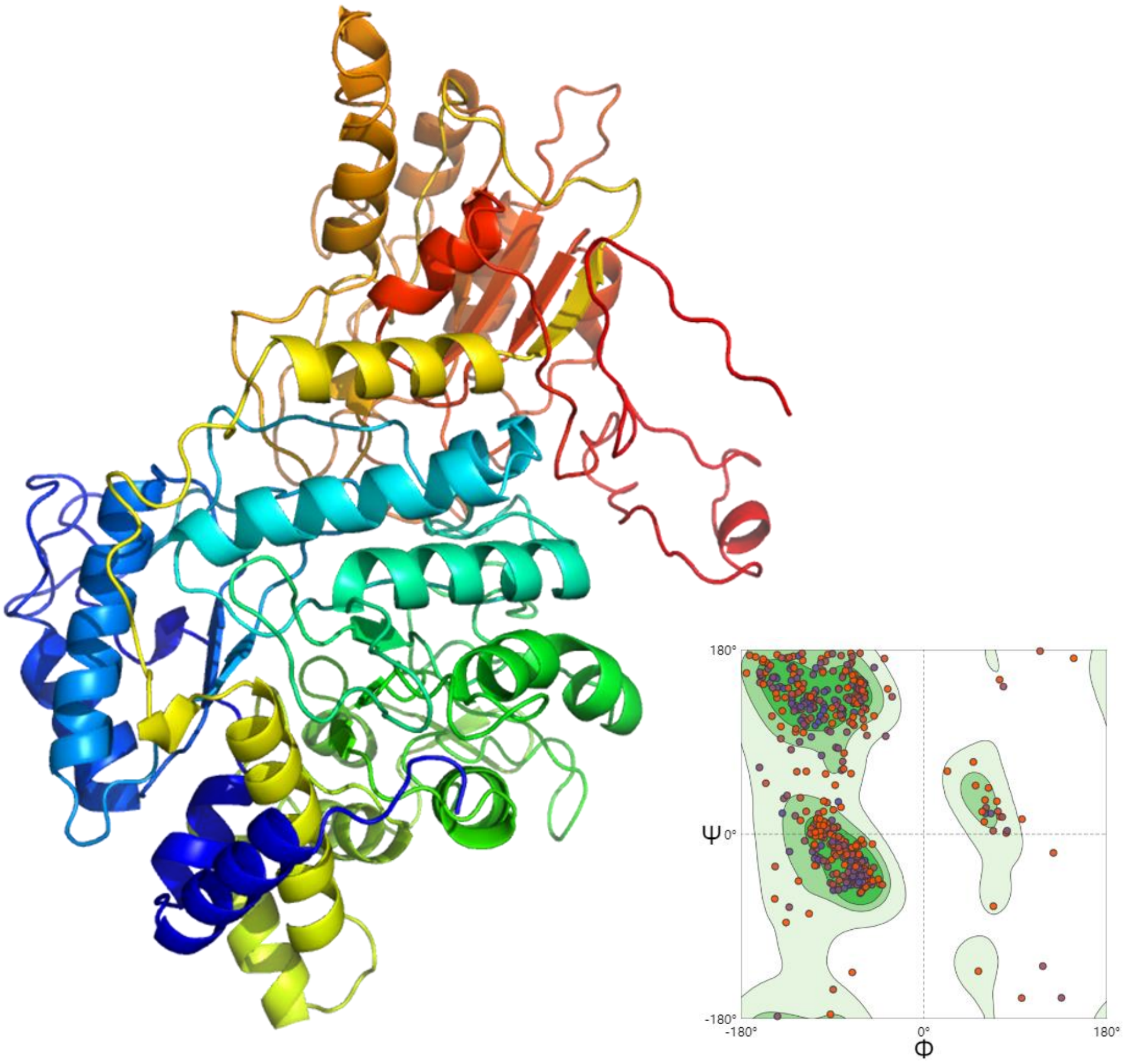

Fig. S15 Swiss-model online tool was used to predict the structure of *Pl*GH3 using 5JP0 as a template. The modeling results show that *Pl*GH3 present the typical (*β/α*) 8 barrel of the GH3 family. Structure assessment by Ramachandran Plots, most amino acid residue is credibles fall in the permitted region, which indicates that the simulation results are credible.

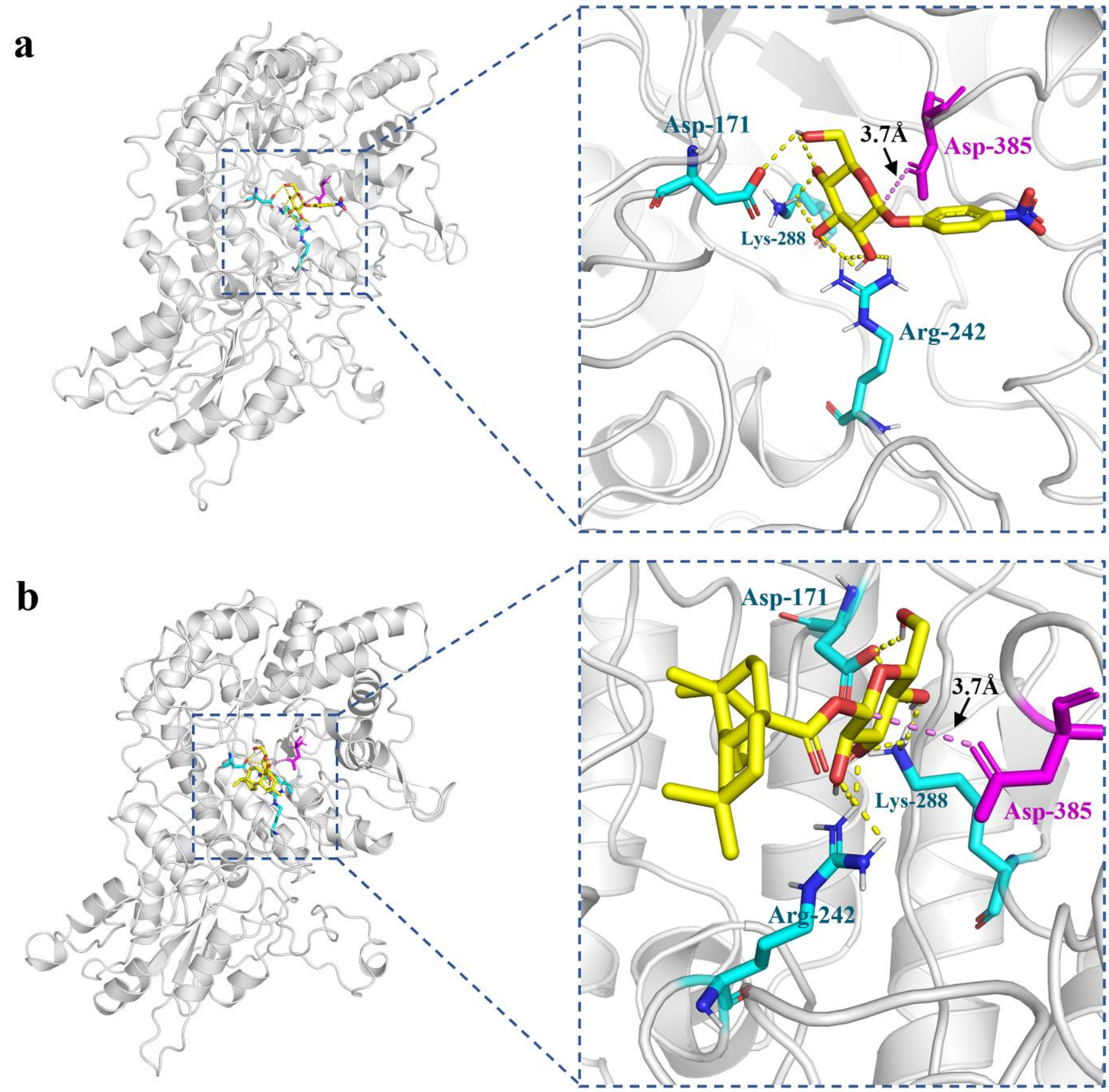

Fig. S16 The molecular docking results of *p*NPG (**a**) and the virtual compound with a structure of perhydrophenanthrene esters (**b**). The amino acid residue Asp385 as a nucleophile is marked as magentas, the distance from the anomeric carbons of the glucose group shown as 3.7 Å, respectively, the key amino acid residue that creates hydrogen bonding with hydroxyl groups on glucose is labeled cyan blue, the H-bonds are marked as yellow.

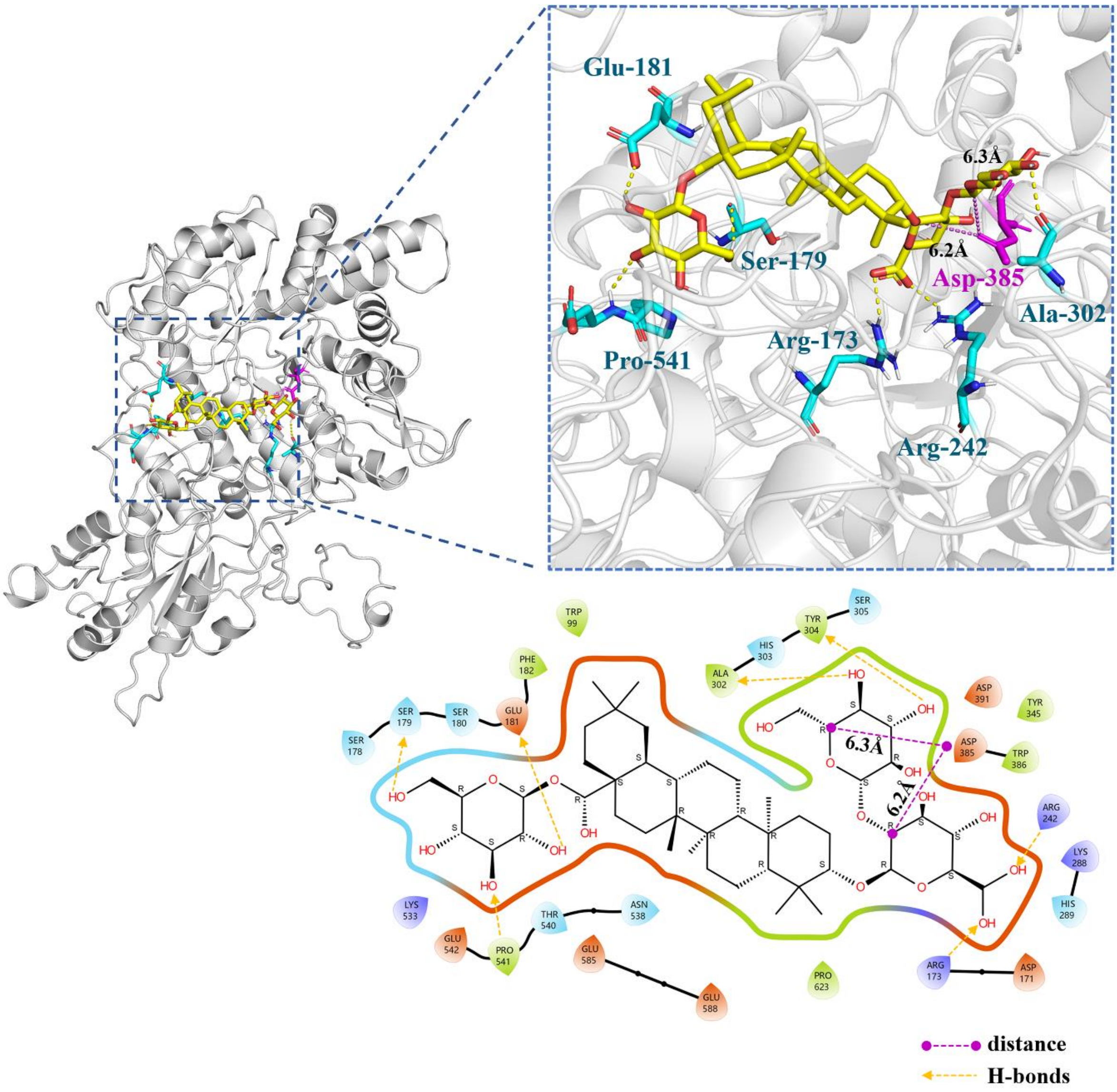

Fig. S17 The molecular docking results of Ginsenoside Ro and *Pl*GH3 on unstable state. The key amino acid residue Asp385 as a nucleophile is marked as magentas, the distance from the anomeric carbons of the glucose group and glucuronic acid group is 6.2 Å and 6.3 Å, respectively, the key amino acid residue that creates hydrogen bonding with hydroxyl groups on glucose is labeled cyan blue, the H-bonds are marked as yellow.

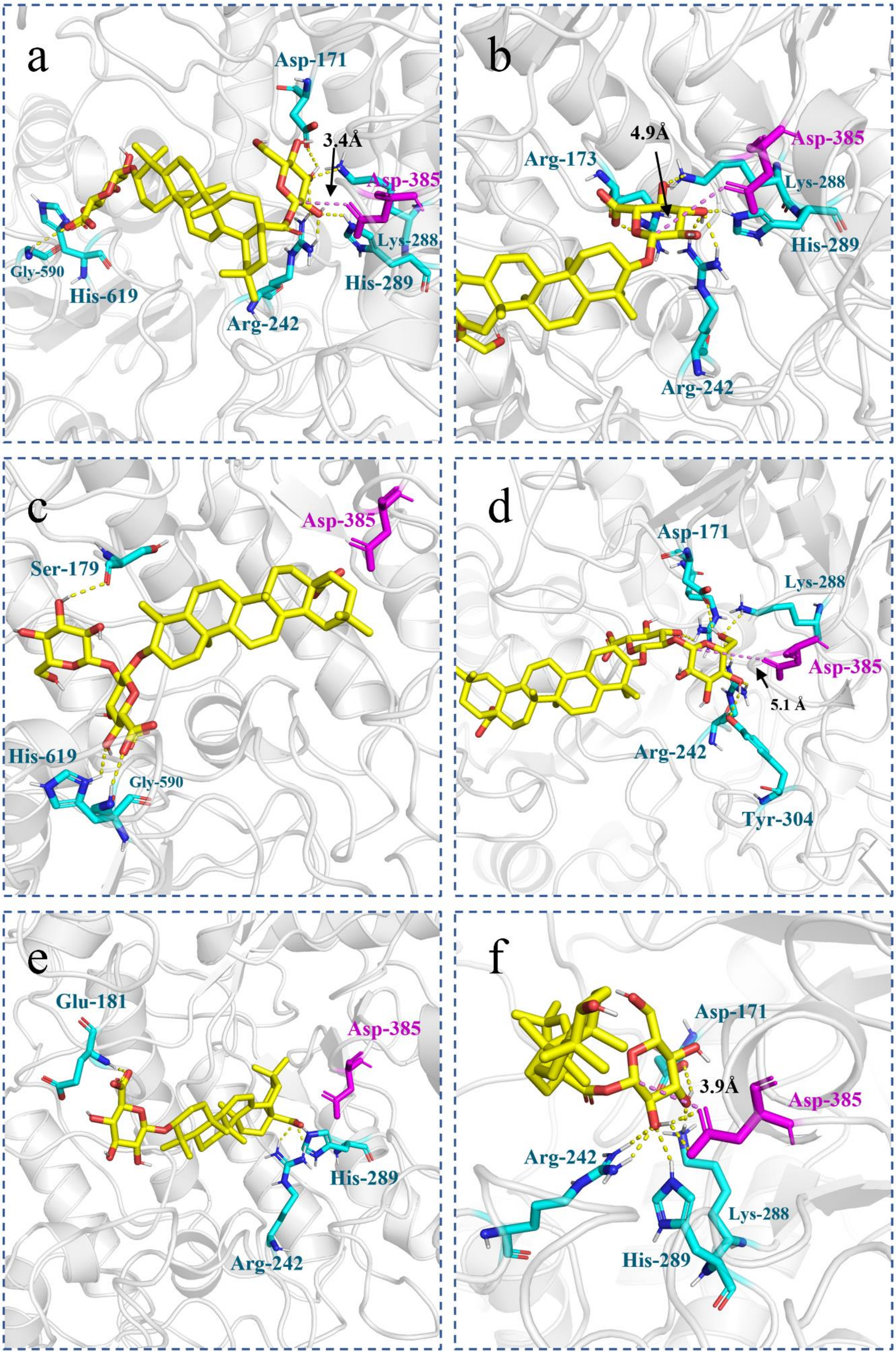

Fig.S18 Molecular docking results of different compounds and *Pl*GH3. The ligands are shown in yellow, Asp385 as a nucleophile is marked as magentas, the key amino acid residue that creates hydrogen bonding with hydroxyl groups on glucose is labeled cyan blue. **a**) the dominant conformation of C-IVa; **b**) the inferior conformation of C-IVa; **c**) the dominant conformation of ZR1; **d**) the dominant conformation of ZR1; **e**) the conformations of CE; **f**) the conformations of oleanolic acids

The amino acid sequence of *Pl*GH3

MRNHTLDTINKTEETVRYVQNPGGPTLGYSEESGVGIIEQDGLFFKDLSRDGKLDNYEDWRLTPEERAKDLASK
MTVEQIAGLMLYSRHQSIPALSSGWFAGTYGGKTYEESGAKPWELTDEQIAFLTKDHVRHVLVTTVESPEVAAR
WNNKIQAFAEGTGLGIPANNSSDPRHASDSSSEFNAGAGGHISMWPETLGLAATFDPEITKKFGMIASREYRA
LGLATALSPQIDIATEPRWFRFNGTFGEDSKLAADMARAYVDGFQTSEGEREIADGWGYDSVNAMVKHWPG
GGSGEAGRDAHYSCGKYAVYPGNNFDEHLVPFTEGAFKLDGKTGKASAVMPYYTISLGQDTVNGENVGNSY
NSYLIRDLLRGKYGYDGVVCTDWMITADVSGPKDSFLSGKPWGVEDLTVGERHYKLQMAGVDQFGGNNEIE
PVLEAYKIGVREHGEAYMRERFEQSAVRLLKNMFRLGLFENPYLDPQESATLVGNPEFMREGYEAQLKSIVMLK
NKNGVLPLRAKSKVYIPKRFLPPGKDWFGNPTPESYDYPVNLEVVSKYFEVTDQPDEAEFGLVFITSPKSGTGYS
QEDEERGGNGYVPISLQYKPYTAEHAREISLAGDEHGNEPRNRSYKGKTVIPHNTTDLNMVLETKEKMKGKPVI
VSMLLCNPTVVSEFEAEVDAILANFGVQDQAMMEVLTGAAEPSGLLPMQMPAHMRTVEEQLEDVAHDMEC
HVDSEKHVYDFAFGMNWGGVIEDERTKRYRRS

The base sequence of *Pl*GH3

ATGAGAAACCATACTTTAGATACGATTAATAAGACAGAAGAAACCGTTCGATATGTACAAAATCCCGGC
GGCCCCACGCTGGGCTACAGCGAGGAATCGGGCGTGGGCATCATCGAGCAGGACGGCTTGTTCTTCAA
GGATTTAAGCCGTGACGGCAAGCTGGACAACTATGAGGACTGGCGGCTGACGCCGGAGGAGCGGGCG
AAAGACCTGGCCTCGAAAATGACGGTCGAGCAGATTGCCGGCCTGATGCTGTACAGCCGCCATCAGTCG
ATTCCCGCGCTCAGTAGCGGCTGGTTTGCAGGCACGTACGGCGGGAAGACGTATGAGGAGAGCGGAGC
GAAGCCCTGGGAACTGACCGATGAGCAGATCGCATTTTTGACCAAAGACCATGTGCGGCACGTGCTTGT
AACCACGGTGGAAAGCCCGGAGGTCGCGGCGCGCTGGAACAATAAAATCCAGGCGTTTGCCGAAGGCA
CCGGTCTCGGGATTCCGGCGAACAACAGCTCCGATCCCCGGCACGCTTCGGATTCAAGCTCCGAATTCA
ACGCGGGTGCGGGCGGCCATATCTCCATGTGGCCCGAGACGCTGGGCCTAGCGGCGACCTTCGATCCG
GAGATCACGAAGAAGTTCGGGATGATCGCTTCCCGGGAATATCGCGCGTTAGGGCTGGCAACCGCCCT
GTCTCCGCAAATCGATATCGCCACGGAGCCGCGCTGGTTCCGGTTTAACGGCACGTTCGGCGAAGATTC
GAAGCTCGCCGCCGATATGGCCCGCGCTTATGTCGACGGCTTCCAGACTTCCGAAGGCGAACGGGAAA
TCGCCGACGGTTGGGGTTACGACAGCGTGAATGCGATGGTGAAGCATTGGCCGGGAGGAGGCTCGGGC
GAGGCCGGAAGGGACGCCCATTACAGCTGCGGGAAGTATGCGGTGTATCCGGGCAACAACTTTGACGA
GCATTTGGTACCTTTTACTGAAGGGGCATTCAAGCTGGACGGCAAAACAGGGAAGGCGTCAGCCGTCAT
GCCGTATTACACGATCTCCCTCGGCCAGGACACCGTAAACGGCGAAAATGTCGGCAACTCCTATAACTC
GTACCTGATTCGGGATTTGCTGCGCGGGAAATACGGGTATGACGGCGTCGTATGCACGGACTGGATGAT
CACGGCCGACGTCTCCGGTCCCAAGGATTCTTTTCTGAGCGGAAAACCATGGGGCGTGGAGGATTTGAC
CGTGGGCGAGCGCCACTACAAGCTGCAAATGGCTGGCGTTGACCAATTCGGCGGCAATAATGAGATCG
AGCCGGTGCTGGAGGCTTACAAGATCGGGGTTCGCGAGCACGGTGAAGCCTATATGCGGGAACGCTTC
GAGCAATCGGCCGTCCGGCTGCTGAAAAATATGTTCCGCCTCGGCTTGTTTGAGAATCCGTACCTCGACC
CACAGGAGAGTGCCACACTGGTCGGGAACCCCGAATTTATGCGGGAAGGTTACGAAGCACAGCTTAAA
TCGATCGTCATGCTCAAAAACAAAAACGGGGTGCTCCCGCTTCGCGCGAAAAGCAAGGTTTACATCCCG
AAACGTTTTCTTCCGCCGGGAAAAGACTGGTTCGGCAATCCGACGCCGGAGAGCTATGATTATCCGGTC
AACCTGGAGGTTGTCTCGAAATATTTCGAAGTCACCGACCAACCGGACGAAGCGGAATTCGGCCTTGTC
TTTATCACATCACCGAAGTCCGGCACCGGCTACAGCCAAGAGGACGAGGAGCGGGGCGGGAACGGTTA
TGTGCCGATCAGCCTGCAGTACAAGCCGTATACGGCGGAGCATGCACGGGAAATCAGCCTGGCCGGCG
ACGAACACGGGAATGAGCCGCGAAATCGTTCTTATAAAGGAAAAACCGTCATTCCGCATAATACGACGG
ATTTAAACATGGTGCTGGAGACGAAGGAGAAAATGAAAGGCAAACCCGTCATCGTCTCCATGCTGTTGT
GCAACCCCACGGTCGTTTCGGAATTTGAAGCGGAAGTGGACGCCATTCTGGCGAACTTCGGCGTTCAGG
ATCAGGCGATGATGGAGGTATTGACGGGAGCAGCGGAGCCGTCCGGTCTGCTGCCGATGCAAATGCCC
GCCCATATGCGCACCGTCGAAGAGCAGTTGGAAGATGTCGCGCACGATATGGAATGCCATGTCGATTCG
GAGAAGCATGTATATGACTTTGCTTTCGGGATGAACTGGGGCGGCGTGATCGAGGATGAGCGAACGAA
GAGATACCGTAGAAGC*
